# Overexpression of the Wheat Aquaporin Gene, *TaAQP7*, Enhances Drought Tolerance in Transgenic Tobacco

**DOI:** 10.1371/journal.pone.0052439

**Published:** 2012-12-20

**Authors:** Shiyi Zhou, Wei Hu, Xiaomin Deng, Zhanbing Ma, Lihong Chen, Chao Huang, Chen Wang, Jie Wang, Yanzhen He, Guangxiao Yang, Guangyuan He

**Affiliations:** Genetic Engineering International Cooperation Base of Chinese Ministry of Science and Technology, Chinese National Center of Plant Gene Research (Wuhan) HUST Part, Key Laboratory of Molecular Biophysics of Chinese Ministry of Education, College of Life Science and Technology, Huazhong University of Science and Technology (HUST), Wuhan, China; RIKEN Plant Science Center, Japan

## Abstract

Aquaporin (AQP) proteins have been shown to transport water and other small molecules through biological membranes, which is crucial for plants to combat stress caused by drought. However, the precise role of *AQPs* in drought stress response is not completely understood in plants. In this study, a *PIP2* subgroup gene *AQP*, designated as *TaAQP7*, was cloned and characterized from wheat. Expression of TaAQP7-GFP fusion protein revealed its localization in the plasma membrane. TaAQP7 exhibited high water channel activity in *Xenopus laevis* oocytes and *TaAQP7* transcript was induced by dehydration, and treatments with polyethylene glycol (PEG), abscisic acid (ABA) and H_2_O_2_. Further, *TaAQP7* was upregulated after PEG treatment and was blocked by inhibitors of ABA biosynthesis, implying that ABA signaling was involved in the upregulation of *TaAQP7* after PEG treatment. Overexpression of *TaAQP7* increased drought tolerance in tobacco. The transgenic tobacco lines had lower levels of malondialdehyde (MDA) and H_2_O_2_, and less ion leakage (IL), but higher relative water content (RWC) and superoxide dismutase (SOD) and catalase (CAT) activities when compared with the wild type (WT) under drought stress. Taken together, our results show that *TaAQP7* confers drought stress tolerance in transgenic tobacco by increasing the ability to retain water, reduce ROS accumulation and membrane damage, and enhance the activities of antioxidants.

## Introduction

In plants, water movement is an extremely important process and is controlled by the apoplastic and symplastic pathways. The latter is more efficient in regulating water transport across the membranes when a plant is in stress [1]–[3]. In the symplastic pathway, aquaporin (AQP) proteins, that form a large family in the major intrinsic protein (MIP) superfamily, play an important role. AQPs are known to transport water and other small molecules through biological membranes and many *AQP* genes have been identified from different plant species [4] including 35 from Arabidopsis [5], 36 from maize [6] and 33 from rice [7]. Compared to other species, little is known about the *AQPs* in wheat because of the unavailability of its complete genome sequence and the allohexaploid nature of its genome.

Many studies have shown that environmental stresses factors such as salt, drought and cold can upregulate *AQPs* and the transgenic approaches established that overexpression of some *AQPs* could improve the plant tolerance to abiotic stress [4], [8]–[14]. In addition, AQPs activitiy may be directly regulated by phosphorylation, which in turn can be influenced by a number of stimuli, including abiotic stress [15]–[18], phytohormones [19] and H_2_O_2_ [17]. Abscisic acid (ABA) is a well-recognized mediator of water stress responses and its exogenous application is known to enhance root hydraulic conductivity in sunflower and maize [20], [21]. Although several isoforms of AQPs facilitate H_2_O_2_ transport across the tonoplast and plasma membrane [19], [22], a number of studies have revealed that H_2_O_2_ in turn could alter the phosphorylation state of AQPs by changing the AQPs structure that leads to the closure of water channels. In addition, H_2_O_2_ can also cause AQP internalization resulting in the downregulation of plant water transport [17], [23]–[26]. Notably, the activities of AQPs were higher in chilling-tolerant genotype than in chilling-sensitive genotype due to less oxidative damage to membranes supported by less IL and H_2_O_2_ content in chilling-tolerant genotype during chilling stress [17]. However, the role of AQPs in enhancing the antioxidant system that relieves membrane damage under drought stress is unclear.

Production of wheat, a global staple crop is constrained by multiple environmental stress factors, such as drought, salinity and extreme temperature. An understanding of the molecular mechanisms underlying the response to abiotic stress responses is necessary for genetic improvement of stress tolerance in wheat. Although *AQP* genes respond to various stresses, their exact role in abiotic stress tolerance remains unclear. In the present study, we characterized a wheat *AQP*, *TaAQP7*, and enhanced drought stress tolerance in transgenic tobacco by maintaining better water status, reducing H_2_O_2_ accumulation and membrane damage via enhancing the activities and expression of superoxide dismutase (SOD; EC 1.15.1.1) and catalase (CAT; EC 1.11.1.6).

## Results

### *TaAQP7* Encodes a PIP2 Subgroup of AQP in Wheat

The cDNA of *TaAQP7* (GenBank ID: HQ650109), was amplified by RACE-PCR using mRNA isolated from the leaves of wheat seedlings. The full-length *TaAQP7* cDNA has 1019 bp with a 861 bp open reading frame (ORF) that translates into the TaAQP7 protein with 286 amino acids and a predicted molecular mass of 30.36 kDa. Blastx analysis showed that *TaAQP7* shared a high degree of sequence similarity with *AQPs* from other plant species: 99% sequence identity with *HvPIP2-1* from *Hordeum vulgare*, 94% with *OsPIP2-2* from *Oryza sativa*, and 91% with *ZmPIP2-2* from *Zea mays*. The predicted TaAQP7 protein contains six putative transmembrane α-helices, a highly conserved amino acid sequence ‘HINPAVTFG’, and two ‘NPA’ motifs [27] (Fig. S1). In addition, it contained the conserved sequence (R/K)DYX(E/D)PP(P/R)X_3–4_(E/D)XXELXXWSF(Y/W)R that is found in all PIP members [27]. A phylogenetic tree was constructed (Fig. S2) based on the amino acid sequence alignment of plant AQPs with Arabidopsis, rice and wheat sequences obtained from GenBank. This showed that on an evolutionary timescale, TaAQP7 was close to PIP2 subfamily suggesting that the wheat *TaAQP7* obtained in this study is a member of the *PIP2* subfamily.

### Expression of *TaAQP7* in *Xenopus laevis* Oocytes Enhances Water Permeability

**Figure 1:**
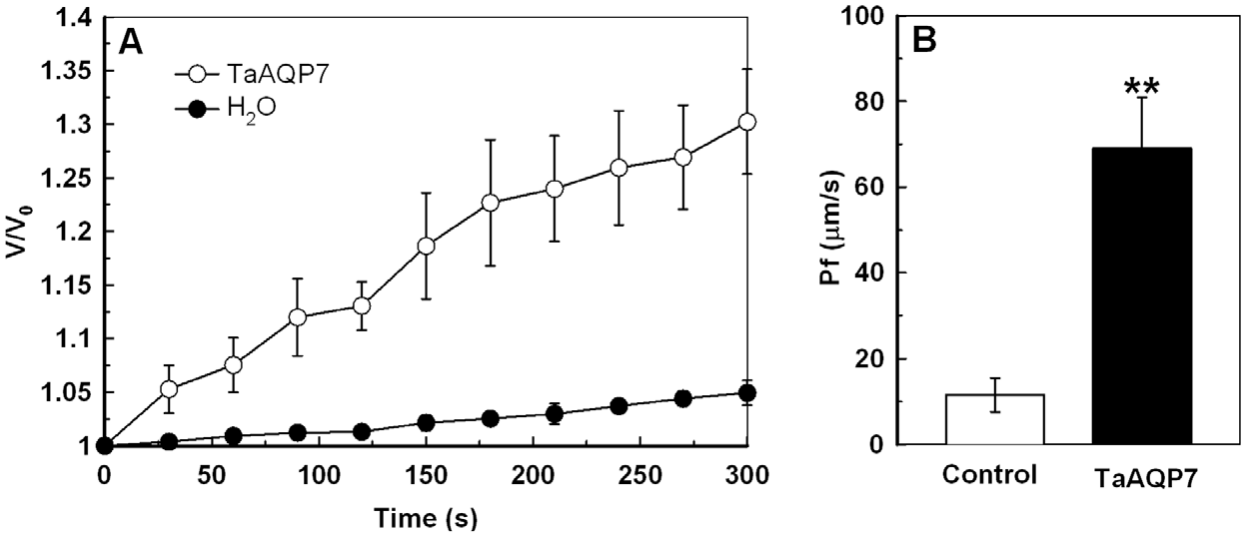
Water channel activity test of TaAQP7. (A) The swelling rates of *Xenopus laevis* oocytes injected with cRNA encoding TaAQP7 or water (as negative control). The rate of oocyte swelling upon immersion in hypo-osmotic medium is plotted as V/V_0_ versus time, where V is the volume at a given time point and V_0_ is the initial volume. (B) Osmotic water permeability coefficient (Pf) of oocytes injected with cRNA encoding TaAQP7 or water. The Pf values were calculated from the rate of oocyte swelling. Vertical bars indicate ±SE of three replicates on one sample (Each replicate contains three oocytes). Asterisks indicate significant difference between oocytes injected with cRNA encoding TaAQP7 and water (*p<0.05; **p<0.01). Three biological experiments were performed, which produced similar results.

To determine whether the *TaAQP7* is a functional *AQP*, water channel activity of the protein was assayed in *Xenopus laevis* oocytes. Two days after cRNA or water injection, the rate of change in cell volume (Fig. 1A) and the osmotic water permeability coefficient (Pf) (Fig. 1B) were calculated in osmotic solution. Oocyte swelling in *TaAQP7*-expressing oocytes and controls are shown in Video S1. The rate of change in cell volume was higher in *TaAQP7*-expressing oocytes than in water-injected oocytes after osmotic treatment. Oocytes expressing *TaAQP7* showed 6-fold higher Pf than the water-injected oocytes, suggesting that *TaAQP7* is a functional *AQP* with high water channel activity.

### *TaAQP7* is Ubiquitously Expressed in Wheat Tissue

*AQPs* not only mediate water transport across plant membranes, but also play a significant role in the different tissues of plants during unfavorable environmental conditions [27]. To determine the expression patterns of *TaAQP7* in different wheat tissues, real-time quantitative polymerase chain reaction (qRT-PCR) was carried out with mRNAs isolated from different tissues as templates (Fig. S3). The results showed *TaAQP7* expression in all organs including root, stem, leaf, stamen, pistil and lemma with higher expression levels in root, stem and leaf.

### Dehydration Stress Upregulates *TaAQP7* Expression in Wheat Seedlings

**Figure 2:**
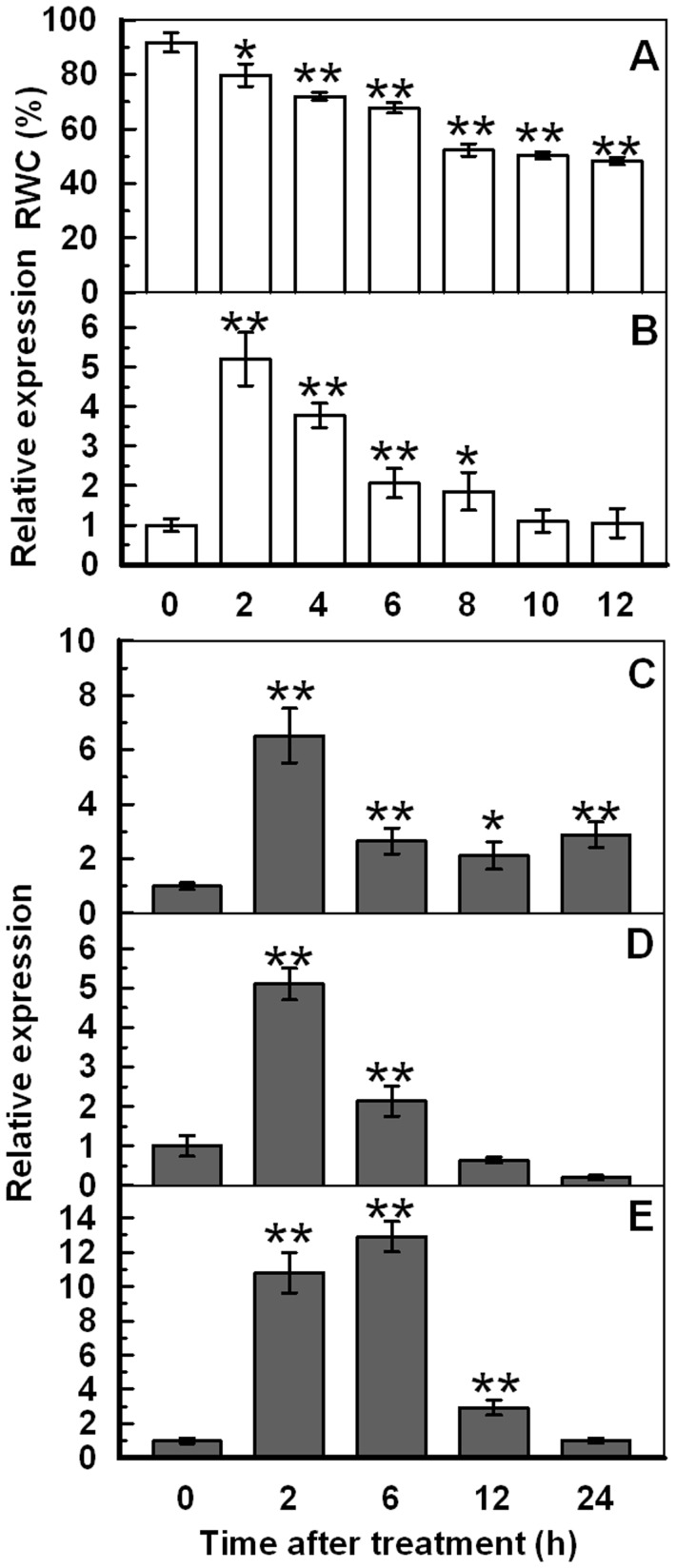
RWC and expression of *TaAQP7* in wheat seedlings with different treatments. Ten-day-old wheat seedlings were subjected to 12 h dehydration treatment and the wheat leaves were sampled to measure RWC (A) and the expression of *TaAQP7* (B). Ten-day-old wheat seedlings were treated with 20% of PEG6000 (C), 100 µM ABA (D), and 10 mM H_2_O_2_ (E) and leaves were sampled within 24 h to extract RNA for qRT-PCR analysis. The relative fold difference in mRNA level was calculated using the 2^–ΔΔCt^ formula with *TaActin* as internal control. The mRNA fold difference was relative to that of distilled water treated samples used as calibrator. Vertical bars indicate ±SE of four replicates on one sample. Asterisks indicate significant difference between the RWC or relative fold difference in mRNA level (*p<0.05; **p<0.01). When no bar is shown, the deviation is smaller than the symbol. Three biological experiments were performed, which produced similar results.

Relative water content (RWC) is a specific tool for the measurement of drought tolerance and gives a credible evaluation of the plant water status [28]. In fact, RWC estimates the deficit in water level in a given time, which is used to reflect dehydration stress [29]. To investigate the relationship between dehydration stress and expression of *TaAQP7*, RWC was measured in untreated wheat seedlings and dehydration stress induced seedlings at different times after the treatment. In parallel, changes in *TaAQP7* expression were also analyzed in these samples by qRT-PCR (Fig. 2). Compared to the well hydrated control, seedlings under dehydration stress displayed lower RWC when the duration of dehydration was increased from 2 to 12 h (Fig. 2A). qRT-PCR showed that the expression level of *TaAQP7* increased by 5.2-fold within 2 h of dehydration stress but reduced to normal levels 12 h after dehydration treatment (Fig. 2B). These results indicated that even a slight water stress could rapidly increase the expression of *TaAQP7*.

### TaAQP7 is Upregulated in Response to Polyethylene Glycol (PEG), ABA and H_2_O_2_ Treatments

We studied the expression of *TaAQP7* for 24 h after treatments with polyethylene glycol (PEG), ABA, methyl jasmonate (MeJA), salicylic acid (SA), auxin and H_2_O_2_. After PEG treatment, the *TaAQP7* transcripts increased 6.5-fold in the first 2 h followed by a decrease and the overall expression of *TaAQP7* remained higher in treated plants when compared to controls (Fig. 2C). The expression levels of *TaAQP7* also increased to a maximum at 2 h (5.1-fold) after ABA treatment and at 6 h (12.9-fold) with H_2_O_2_ treatment (Fig. 2D and E). MeJA treatment also increased the expression of *TaAQP7* continuously, while auxin and salicylic acid treatments inhibited *TaAQP7* expression (Fig. S4). These data indicate that *TaAQP7* is induced by PEG, ABA, H_2_O_2_ and MeJA and a similar gene expression pattern is observed during PEG, ABA, H_2_O_2_ treatments.

### ABA is Involved in the Induction of *TaAQP7* by PEG Treatment

**Figure 3:**
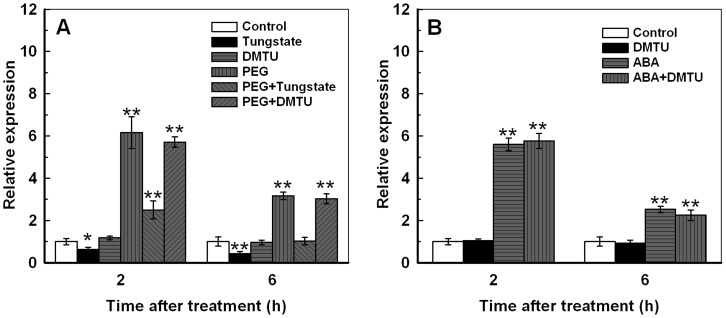
Effects of inhibitors of signaling molecules on the *TaAQP7* transcripts under PEG and ABA treatments. A: Effects of pretreatment with inhibitors of ABA biosynthesis and scavenger H_2_O_2_ on the expression of *TaAQP7* in the leaves of wheat seedlings exposed to PEG. The plants were pretreated with distilled water, 1 mM tungstate, and 5 mM DMTU for 2 h and 6 h respectively, and then exposed to 20% PEG6000 for 2 h and 6 h respectively. The treatment of tungstate or DMTU alone was also performed in the experiment. The plants treated with distilled water for 2 h or 6 h were used as control. B: Effects of pretreatment with scavenger of H_2_O_2_ on the expression of *TaAQP7* in the leaves of wheat seedlings exposed to ABA. The plants were pretreated with distilled water, and 5 mM DMTU for 2 h and 6 h respectively, and then exposed to100 µM ABA for 2 h and 6 h respectively. The treatment of DMTU alone was also performed in the experiment. The plants treated with distilled water for 2 h or 6 h were used as control. The y-axis represents the relative fold difference in mRNA level calculated using the 2^–ΔΔCt^ formula with *TaActin* as internal control. The mRNA fold difference was relative to that of distilled water treated samples used as calibrator. Vertical bars indicate ±SE of four replicates on one sample. Asterisks indicate significant difference between the expression of *TaAQP7* under different treatment (*p<0.05; **p<0.01). When no bar is shown, the deviation is smaller than the symbol. Three biological experiments were performed, which produced similar results.

To explore whether the upregulation of *TaAQP7* under PEG treatment involves ABA and H_2_O_2_ signaling, tungstate was chosen as the inhibitor of ABA biosynthesis and dimethylthiourea (DMTU) was chosen as the scavenger of H_2_O_2_ [30], [31]. Plants were pretreated with tungstate and DMTU for 2 h and 6 h followed by PEG treatment. The results clearly show that *TaAQP7* reached a maximum at 2 h (6.3-fold) and 6 h (3.0-fold) after the treatment with PEG (Fig. 3A), consistent with the results in Fig. 2C. Treatment with tungstate reduced the maximum fold increase to 2.8-fold at 2 h and 0.9-fold at 6 h after the PEG treatment, while DMTU had no effect on the upregulation of *TaAQP7* (Fig. 3A). In addition, after treatment with tungstate, the expression of *TaAQP7* was inhibited, while the DMTU treatment had no effect on *TaAQP7* transcript (Fig. 3). H_2_O_2_ plays an important role in signal transduction of various plant cells/tissues under abiotic stress, and evidence suggests the function of H_2_O_2_ down-stream of ABA [32]–[40]. Therefore, we determined the role of H_2_O_2_ in the upregulation of *TaAQP7* expression induced by ABA. First, wheat seedlings were pretreated with DMTU for 2 h and 6 h to stop the production of H_2_O_2_ [30], [40] followed by exposure to ABA treatment for 2 and 6 h. Fig. 3B shows that *TaAQP7* was induced by ABA 2 (6.5-fold) and 6 h (3.5-fold) after treatment, which is in line with the results in Fig. 2D. In addition, pretreatments with H_2_O_2_ scavenger had no effect on the upregulation of *TaAQP7* in the ABA-treated wheat seedlings (Fig. 3B). These results suggest that PEG induced upregulation of *TaAQP7* possibly involves ABA signaling but the upregulation of *TaAQP7* induced by PEG and ABA treatment may not involve H_2_O_2_.

### Generation of Transgenic Tobacco Overexpressing *TaAQP7*

The role of *TaAQP7* in drought stress tolerance was further investigated by generating transgenic tobacco plants overexpressing *TaAQP7* under the control of CaMV 35S promoter. A total of 13 transgenic lines (T_1_) were selected by resistance to hygromycin and confirmed by PCR using primers specific to *TaAQP7* and *GFP* (data not shown). Among the T_1_ lines, three lines (OE6, OE9 and OE13) segregated with a ratio of 3∶1 for hygromycin resistance. In addition, all the three independent transgenic T_2_ line seedlings survived on MS medium containing 40 mg/L of hygromycin. In this experiment, tobacco plants transformed with only the vector served as a negative control and were subjected to the same analysis.

### Overexpression of *TaAQP7* Improves Tolerance of Transgenic Tobacco Plants to Drought Stress

**Figure 4:**
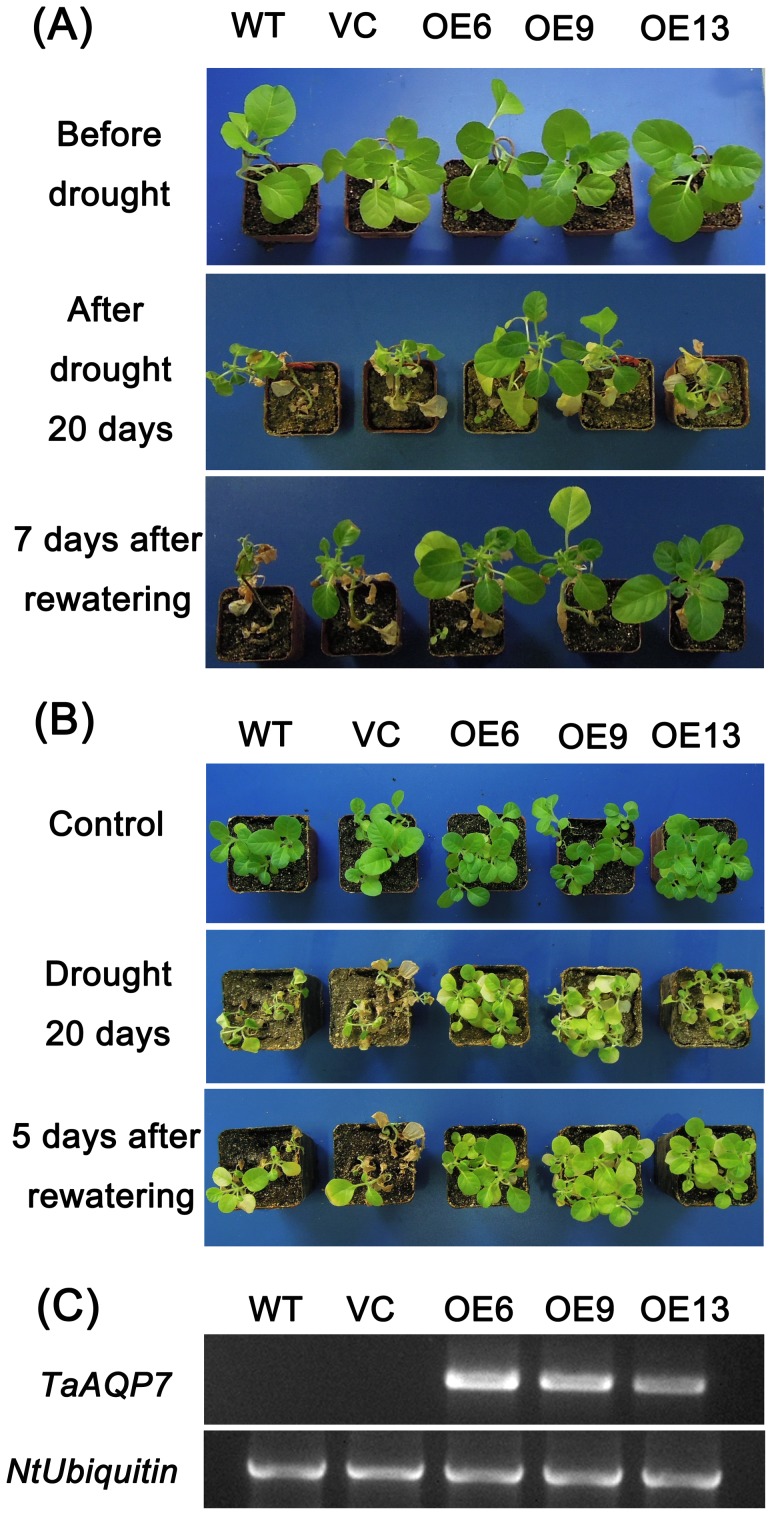
Enhanced drought tolerance in transgenic lines when compared with the WT and VC. The WT, VC and transgenic lines were cultured in MS medium under a 16 h light/8 h dark cycle at 25°C for one week, and then the plants were transplanted to containers filled with a mixture of soil and sand (3∶1) where they were regularly watered for two or five weeks. Six-week-old seedlings of transgenic and WT tobacco plants grown in pots were deprived of water for 20 d and the photos were taken (A). Three-week-old seedlings of transgenic and WT tobacco plants grown in pots were deprived of water for 20 d and the photos were taken (B). The whole two-week-old seedlings of transgenic plants and WT were used to extract RNA to detect *TaAQP7* expression by RT-PCR with *NtUbiquitin* as an internal control (C). Three biological experiments were performed, which produced similar results.

Phenotypes of the transgenic lines, the vector control (VC) and the wild type (WT) under drought stress were recorded. After 20 d of drought stress, leaf wilting was evident in the WT and VC in comparison with the three transgenic lines in both three- and six-week-old tobacco plants (Fig. 4A and B). Five days after providing water, most of the three-week-old VC and WT plants were dead, while most of the transgenic lines survived (Fig. 4B). RT-PCR analysis showed the presence of *TaAQP7* mRNA in the three transgenic lines (OE6, OE9 and OE13) but not in WT and VC (Fig. 4C). Among the transgenic lines OE6 and OE9 had higher *TaAQP7* expression levels. As shown in Fig. 4B, some of the rosette leaves were curled in OE13, while in OE6 and OE9 they remain expanded. Thus, the lines (OE6 and OE9) with higher expression of *TaAQP7* showed an increased tolerance to drought stress, suggesting that the overexpression of *TaAQP7* could improve plants’ tolerance to drought stress.

### Overexpression of *TaAQP7* Enhances the Osmotic Stress Tolerance

**Figure 5:**
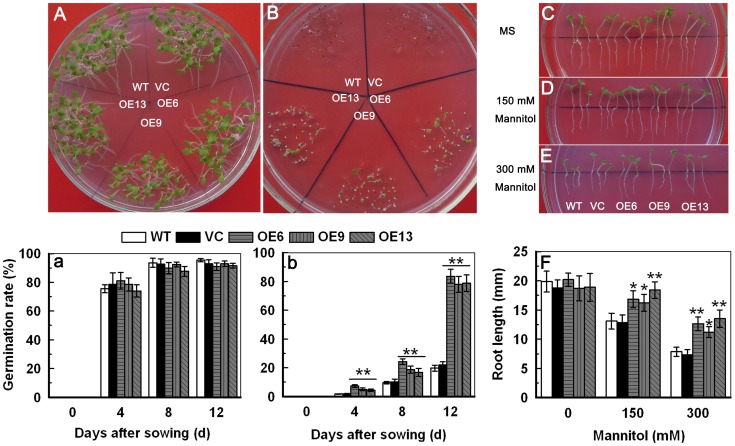
Osmotic tolerance analysis of *TaAQP7*-overexpressing plants. A total of 200 surface-sterilized seeds of each transgenic line, WT or VC germinated on MS medium containing 0 (A, a) or 300 mM (B, b) mannitol for 12 d, and the germination rate was calculated. A, B are photos of the first 12 days after germination on mediums. The WT, VC and transgenic lines were cultured in MS medium under a 16 h light/8 h dark cycle at 25°C for one week, and then the seedlings were transplanted to MS or MS supplied with 150 or 300 mM mannitol for one week. The photographs were taken (C, D, E) and root length was calculated (F). Vertical bars indicate ±SE calculated from four replicates. Asterisks indicate significant difference between the WT and the three transgenic lines (*p<0.05; **p<0.01). Three biological experiments were performed, which produced similar results.

To detect the differences in the germination rates of seeds in transgenic, WT and VC, plants were germinated on MS medium supplemented with 0 and 300 mM mannitol for 12 days. Germination rates were remarkably higher in the transgenic lines (OE6 - 83.60, OE9 - 78.05 and OE13 - 78.83) relative to the WT (19.78) and VC (21.90) on medium with 300 mM mannitol (Fig. 5B and b), while little difference was observed between the transgenic lines and control plants grown on MS medium without mannitol (Fig. 5A and a). Seedlings grown on MS medium alone for 7 days were transferred to MS or MS supplemented with 0, 150 or 300 mM mannitol for 7 days followed by root length measurement. On MS medium alone, all the transgenic plants showed little or no difference in growth compared to WT and VC plants (Fig. 5C and F). On MS containing 150 mM mannitol, longer root length was observed in the transgenic lines OE6 (16.85 mm), OE9 (16.25 mm) and OE13 (18.4 mm) when compared to WT (13.10 mm) and VC (12.85 mm) (Fig. 5D and F). Similarly, on MS containing 300 mM mannitol, the transgenic lines OE6 (12.65 mm), OE9 (11.23 mm) and OE13 (13.50 mm) exhibited longer root length than WT (7.85 mm) and VC (7.35 mm) (Fig. 5E and F). These results indicate that seed germination and root elongation in transgenic tobacco plants overexpressing of *TaAQP7* were acclimatized to osmotic stress.

### Overexpression of *TaAQP7* Improves the RWC and Decreases Malonaldehyde (MDA) and Ion Leakage (IL) under Drought Stress

**Figure 6:**
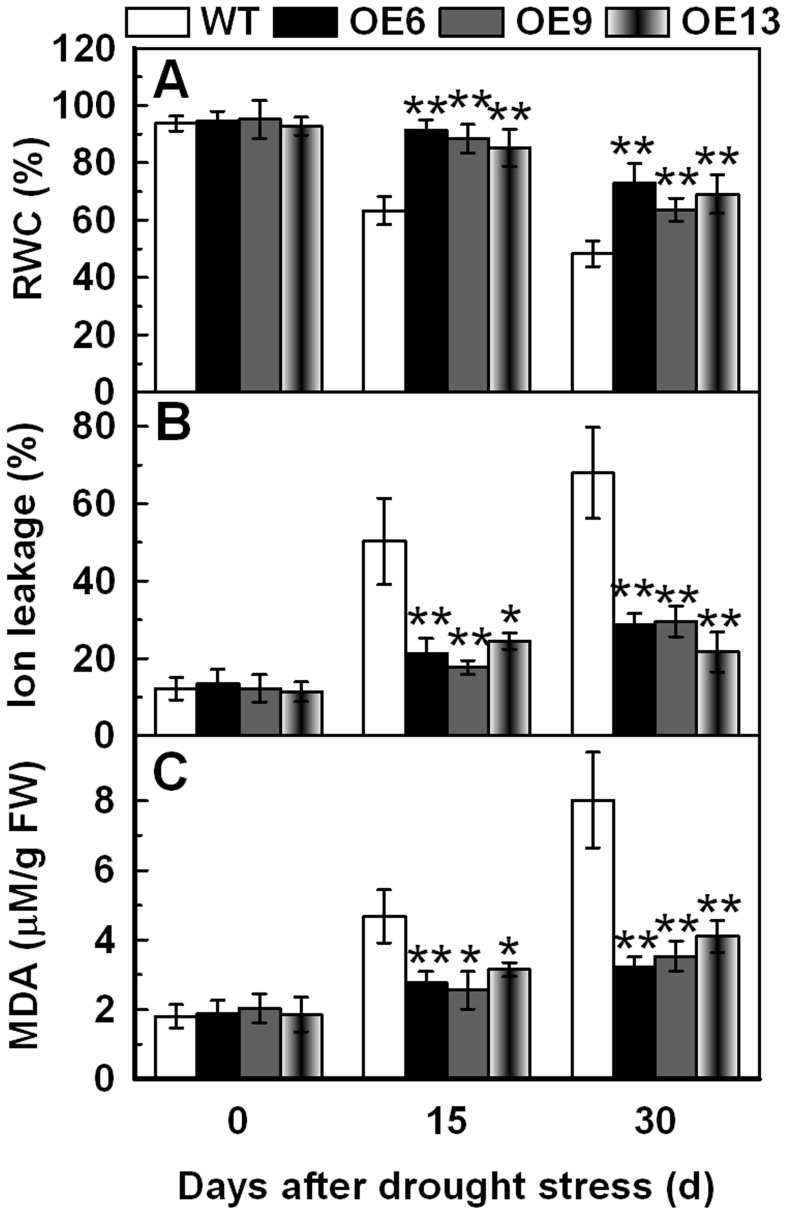
Analysis of RWC, IL and MDA in transgenic lines under drought stress. The WT and transgenic lines were cultured in MS medium under a 16 h light/8 h dark cycle at 25°C for one week, and then the plants were transplanted to containers filled with a mixture of soil and sand (3∶1) where they were regularly watered for two weeks. Three-week-old tobacco plants were deprived of water for 30 d. Tobacco leaves were sampled from WT and transgenic lines under drought stress for 15 d and 30 d to detect RWC (A), IL (B) and MDA (C). Vertical bars indicate ±SD calculated from four replicates. Asterisks indicate significant difference between the WT and the three transgenic lines (*p<0.05; **p<0.01). Three biological experiments were performed, which produced similar results.

Enhanced drought tolerance in transgenic lines compared to the WT led us to observe differences in their physiology. The RWC results show that loss of water in the OE6 (91.3–72.9%), OE9 (88.4–63.7%) and OE13 (85.3–69.1%) transgenic lines was remarkably lesser compared to the WT (63.4–48.3%) when subjected to 15 and 30 d drought stress (Fig. 6A). Ion leakage (IL), an important indicator of membrane injury, was significantly higher in the WT (50.3–68.0%) than in OE6 (21.3–28.7%), OE9 (17.7–29.6%) and OE13 (24.4–21.7%) under drought stress. This suggested that the transgenic plants suffered less membrane damage than WT (Fig. 6B). Malonaldehyde (MDA) is the product of lipid peroxidation caused by reactive oxygen species (ROS), and is in general used to evaluate ROS-mediated injuries in plants [41]. The MDA measurement displayed a pattern similar to the IL and was lower in the transgenic lines relative to the WT under drought stress (Fig. 6C). These physiological indices demonstrated that the transgenic lines were more resistant to drought stress.

### Overexpression of *TaAQP7* Increases the SOD and CAT Activities, and Decreases the H_2_O_2_ Content under Drought/osmotic Stress

**Figure 7:**
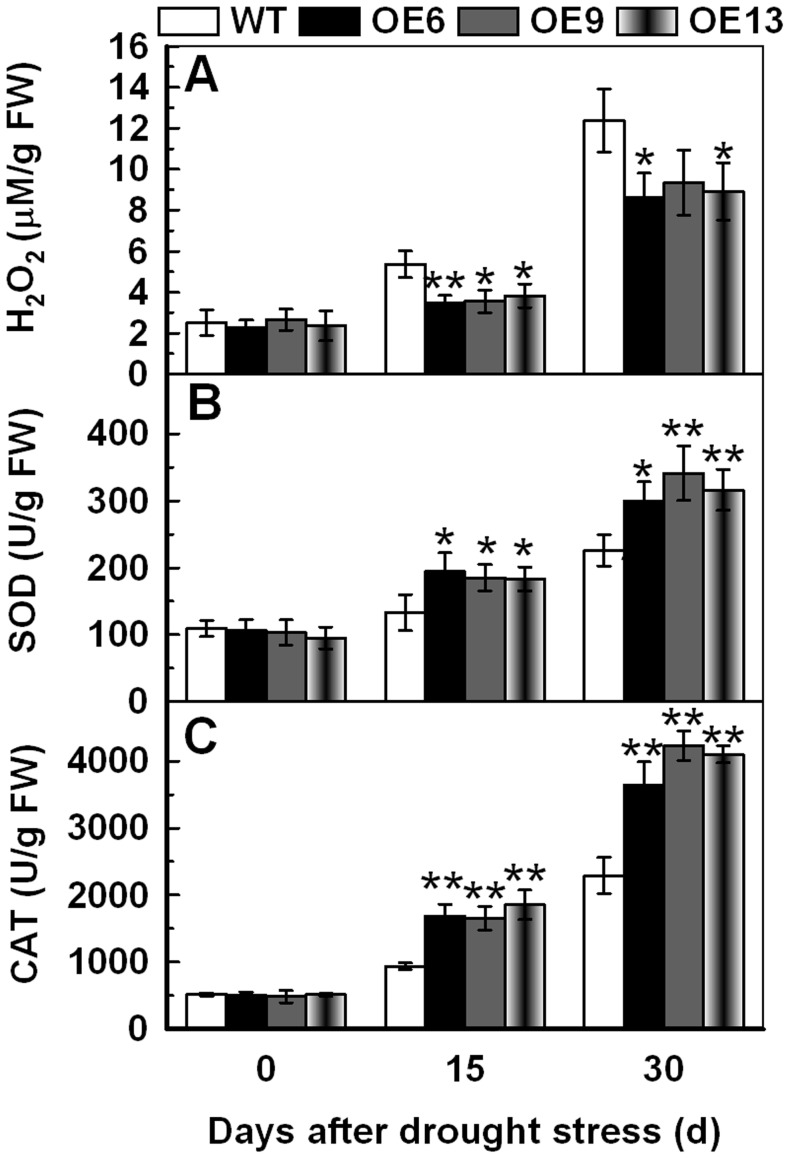
Analysis of H_2_O_2_ content, SOD and CAT activities in transgenic lines under drought stress. The WT and transgenic lines were cultured in MS medium under a 16 h light/8 h dark cycle at 25°C for one week, and then the plants were transplanted to containers filled with a mixture of soil and sand (3∶1) where they were regularly watered for two weeks. Three-week-old tobacco plants were deprived of water for 30 d. Tobacco leaves were sampled from WT and transgenic lines under drought stress for 15 d and 30 d to detect H_2_O_2_ content (A), SOD (B) and CAT (C) activities. Vertical bars indicate ±SD calculated from four replicates. Asterisks indicate significant difference between the WT and the three transgenic lines (*p<0.05; **p<0.01). Three biological experiments were performed, which produced similar results.

The lower ion leakage and MDA level in three transgenic lines imply that they may suffer less membrane damage and lipid peroxidation when compared to WT under drought stress. Therefore, we determined the levels of ROS accumulation in the transgenic and WT plants before and after drought stress. As shown in Fig. 7A, all three transgenic lines had lower H_2_O_2_ content (OE6 3.5–8.6, OE9 3.6–9.3 and OE13 3.8–8.9) than the WT (5.4–12.4) after exposure to drought for 15 and 30 d. In addition, antioxidant enzymes are known to play significant roles in ROS scavenging which inﬂuences the cellular ROS levels. Therefore, the activities of CAT, SOD and peroxidase (POD; EC 1.11.1.7) were measured in leaves of plants before and after drought treatment. After 15 and 30 d drought stress the transgenic lines had significantly higher SOD (OE6 194.3–299.5, OE9 185.4–341.2 and OE13 183.3–316.3) and CAT (OE6 1684.2–3643.4, OE9 1650.8–4230.7 and OE13 1856.7–4104.3) activities than the WT (SOD 133.2–226.3, CAT 935.5–2291.0) (Fig. 7B and C). There was no obvious difference in POD activity between transgenic lines and WT under drought treatment (data not shown).

**Figure 8:**
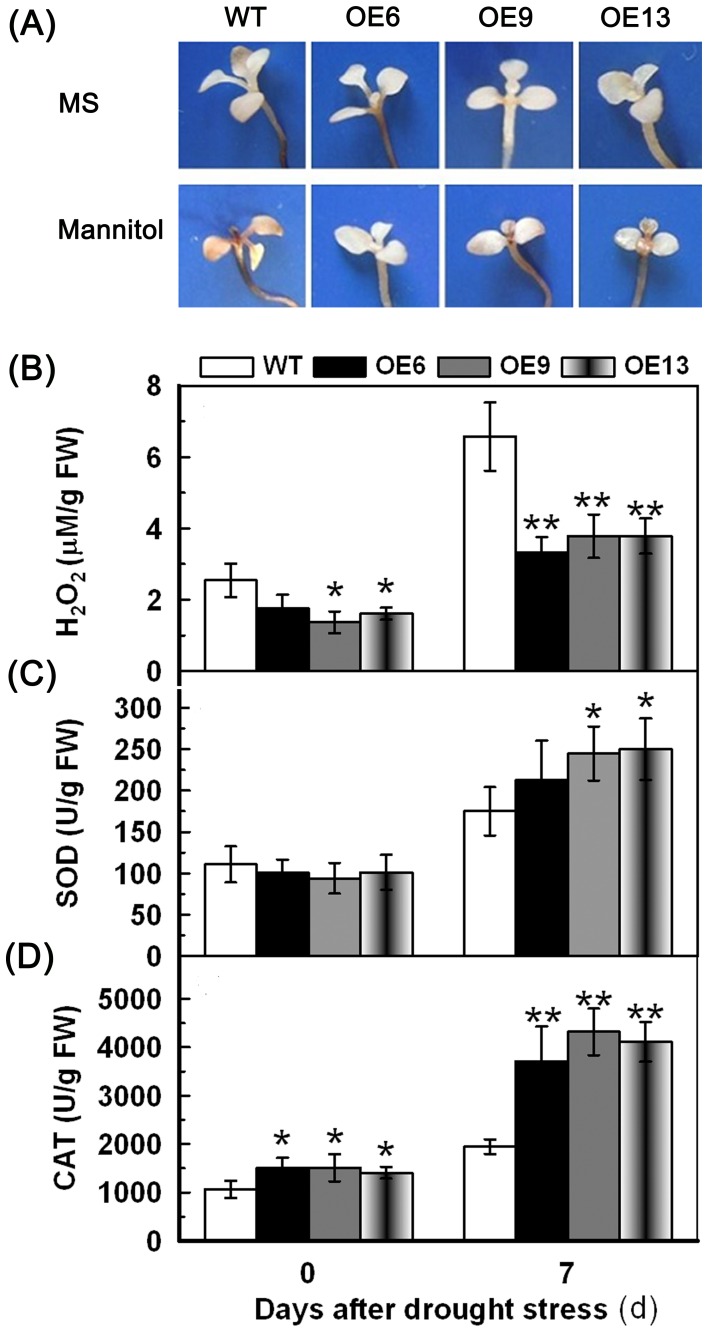
Analysis of H_2_O_2_ accumulation, SOD and CAT activities in transgenic lines under osmotic stress. The WT and transgenic lines were cultured in MS medium under a 16 h light/8 h dark cycle at 25°C for one week, and then the seedlings were transplanted to MS or MS with 300 mM mannitol for one week. The whole seedlings were used to detect H_2_O_2_ accumulation, SOD and CAT activities. (A) In situ detection of H_2_O_2_ by DAB staining of WT and transgenic seedlings. (B) H_2_O_2_ content. (C) SOD activity. (D) CAT activity. Vertical bars indicate ±SD calculated from four replicates. Asterisks indicate significant difference between the WT and the three transgenic lines (*p<0.05; **p<0.01). Three biological experiments were performed, which produced similar results.

To evaluate the content of H_2_O_2_ and the activities of SOD and CAT in WT and transgenic lines under osmotic stress, whole seedlings were used. After 7 d of mannitol treatment, the transgenic lines accumulated lower amounts of H_2_O_2_ than WT as observed by the accumulation of brown pigment (DAB staining) (Fig. 8A), and little difference was observed under normal conditions. The H_2_O_2_ content and, SOD and CAT activities after mannitol treatment showed a pattern similar to the drought stress treatment (Fig. 8B, C and D) suggesting that overexpression of *TaAQP7* reduced H_2_O_2_ accumulation and enhanced SOD and CAT activities under drought/osmotic stress.

### *TaAQP7*-overexpressing Tobacco Plants Exhibit Higher SOD and CAT Activities Relative to WT when they were in the same Water Status under Drought Conditions

**Figure 9:**
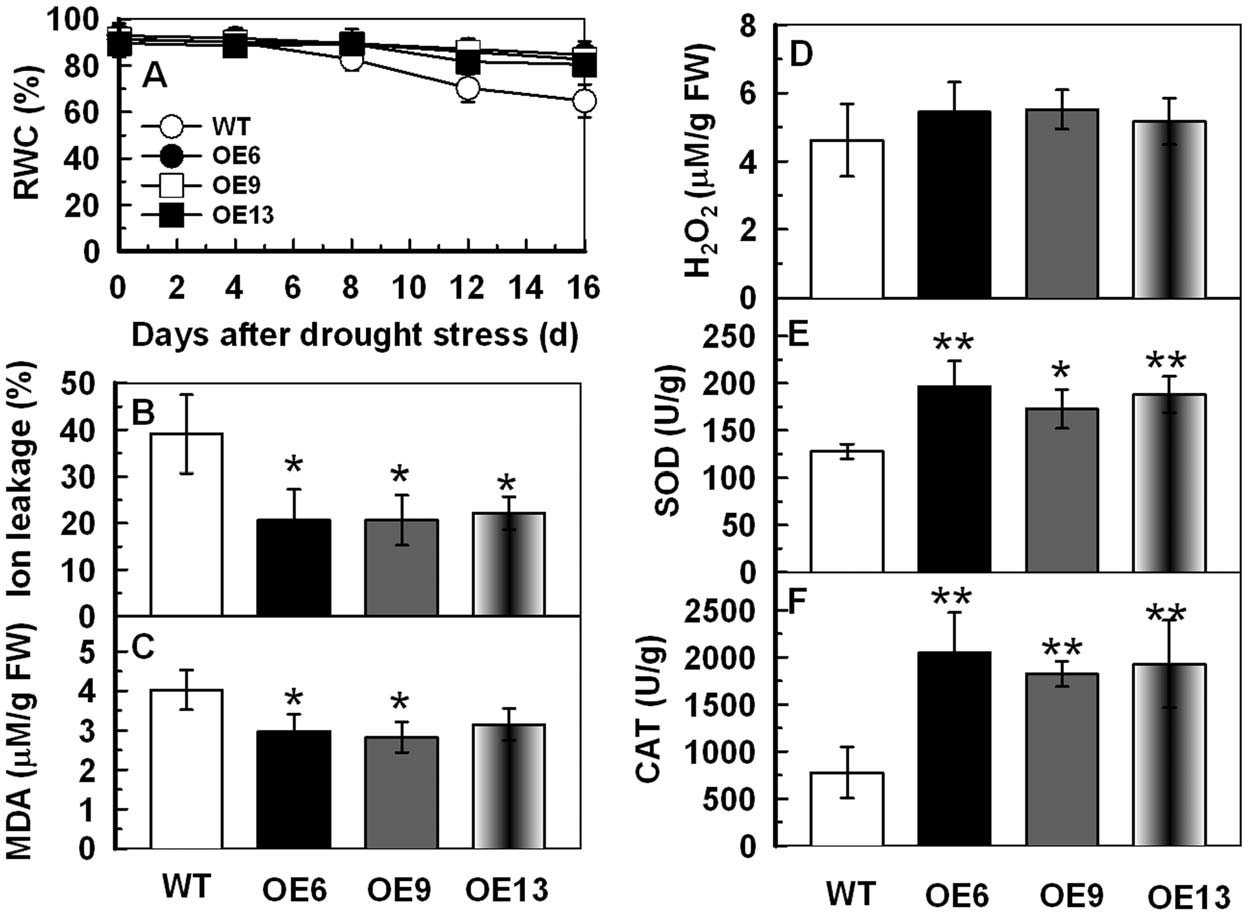
The IL, MDA, H_2_O_2_ content and the activities of SOD and CAT in WT and transgenic lines with the same water status under drought conditions. The WT and transgenic lines were cultured in MS medium under a 16 h light/8 h dark cycle at 25°C for one week, and then the plants were transplanted to containers filled with a mixture of soil and sand (3∶1) where they were regularly watered for two weeks. Three-week-old tobacco plants were deprived of water for 16 d. Tobacco leaves were sampled from WT and transgenic lines under drought stress to detect the RWC (A). When WT with 8 d drought treatment and transgenic lines with 16 d drought treatment, they maintained same water status and then the IL (B), MDA (C), H_2_O_2_ (D), SOD (E) and CAT (F) were measured.

To determine whether the enhancement of antioxidant enzyme activity in the transgenic lines was linked to better water status, all parameters including IL, MDA and H_2_O_2_ levels, and SOD and CAT activities were measured in WT and transgenic plants under the same RWC. After 16 d drought stress, the WT displayed greater decrease in RWC than transgenic plants (Fig. 9A) and the RWC in transgenic lines after 16 d was close to that in WT after 8 d of drought treatment. In addition, MDA and IL (Fig. 9B and C) were lower, SOD and CAT activities were higher (Fig. 9E and F) and no difference was observed in H_2_O_2_ content (Fig. 9D) in the transgenic plants compared to WT under similar water status. These results suggest that the higher activities of SOD and CAT in the transgenic plants are not related to the water status.

### TaAQP7 is Localized in the Plasma Membrane

**Figure 10:**
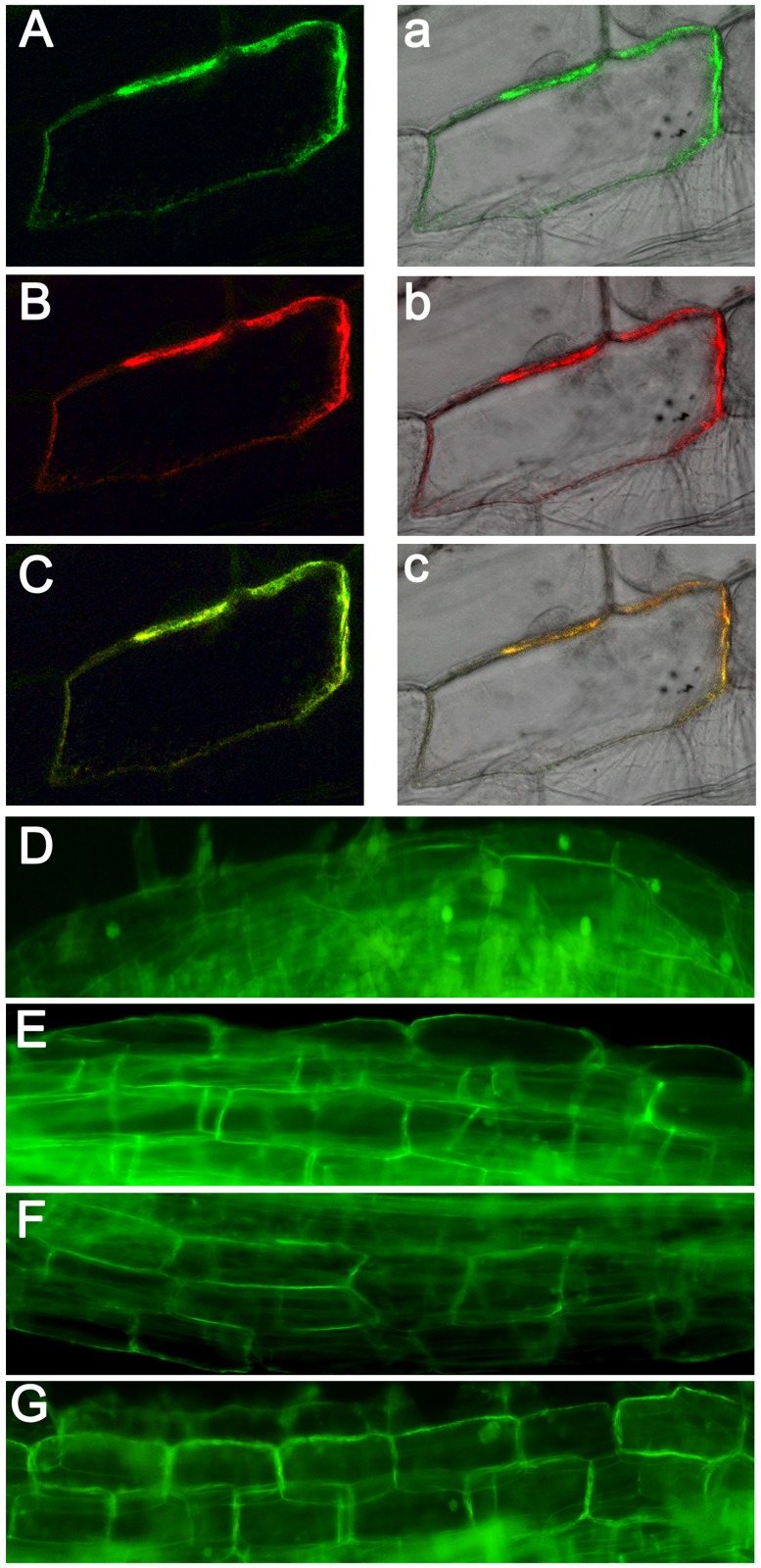
Subcellular localization of TaAQP7 protein. Onion epidermal cells transiently co-transformed with TaAQP7::GFP and pm-rk (Plasma membrane marker). (A, a) Fluorescence image of epidermal cell expressing the p35S-TaAQP7::GFP fusion protein. (B, b) Fluorescence image of epidermal cell expressing the pm-rk. (C, c) Merged fluorescence image of epidermal cell expressing the p35S-TaAQP7::GFP fusion protein and pm-rk marker. Images are dark field (A, B, C), bright field (a, b, c). Microscopy images of tobacco root cortical cells transformed with GFP alone as a control or TaAQP7. (D) Control; (E) OE6; (F) OE9; (G) OE13. Three biological experiments were performed, which produced similar results.

To investigate its cellular localization, the *TaAQP7* ORF was cloned into the pCAMBIA1304-GFP vector downstream of the constitutive promoter, CaMV 35S and upstream of *GFP* to create TaAQP7-GFP fusion construct. The TaAQP7-GFP fusion protein and pm-rk (Plasma membrane marker) were co-expressed in onion epidermal cells by particle bombardment, which is an established method to investigate subcellular localization of GFP-proteins in plant cells [42], [43]. After incubation at 25°C for 24 h, the green fluorescence and red pm-rk were both confined to the plasma membrane (Fig. 10A, B and C). In addition, the subcellular localization of the TaAQP7-GFP fusions in young roots from transgenic tobacco seedlings was observed under a microscope. In the root cells of TaAQP7-GFP-transformed plants (Fig. 10E, F, G), GFP-tagged TaAQP7 protein was mostly localized in the plasma membranes of cells while no ﬂuorescence was observed in the nuclei. In contrast, ﬂuorescence was found in cell nuclei, cytoplasm and plasma membrane in the root cells of plants expressing only GFP (Fig. 10D). Thus, TaAQP7 protein was confirmed to be localized in the plasma membrane of cells.

## Discussion

Although more than 35 *AQP* genes have been identified in wheat genome to date, the function of only a handful of them, such as *TaNIP*, *TdPIP1;1* and *TdPIP2;1* have been characterized [14], [44], [45]. In the present study, an *AQP* cDNA from wheat, designated *TaAQP7*, was cloned and characterized. *TaAQP7* shared a high degree of sequence similarity with *AQPs* from *Hordeum vulgar*, *Oryza sativa*, and *Zea mays*. Amino acid sequence analysis showed that TaAQP7 contains six putative transmembrane α-helices and a conserved sequence which is found in all PIP members (Fig. S1) [27]. The cellular localization studies revealed that TaAQP7 localizes in the plasma membrane (Fig. 10), which is consistent with the characteristics of intrinsic membrane proteins. In addition, on the evolutionary timescale, TaAQP7 displayed a close evolutionary relationship with PIP2 subfamily that is important for the water channel activity (Fig. S2) [12]. Accordingly, *TaAQP7*-overexpressing oocytes exhibited higher Pf than controls (Fig. 1B), suggesting that it is a functional AQP with high water channel activity which is a feature of the PIP2 subfamily. Expression pattern in different tissue indicates that *TaAQP7* is mainly expressed in vegetative tissues (Fig. S3). These data allow us to conclude that TaAQP7 belongs to the PIP2 subfamily of AQP.

Numerous studies have shown that AQPs play an important role in abiotic stress such as salt [4], [9], [14], drought [9], [12] and cold [8], [10]. During the dehydration treatment, even a slight water stress rapidly enhanced the expression of *TaAQP7*, which suggests that the *TaAQP7* gene was very sensitive to dehydration stress (Fig. 2B). Accordingly, PEG treatment could also rapidly upregulate *TaAQP7* (Fig. 2C). The different responses of *AQPs* expression (up/downregulation or no change) to water stress is widely observed in plants. In Arabidopsis, drought treatment rapidly reduced the expression level of *PIP1;5*, *PIP2;2*, *PIP2;3*, and *PIP2;6* transcripts to one-tenth of the level under normal condition [46]. However, *AtPIP1-4* and *AtPIP2-5* transcripts in Arabidopsis leaves were upregulated under drought stress [47]. Thus, the response of AQP family members to drought stress exhibits different patterns. In addition, *AQPs* present in the same species, but in different cultivars, can respond differently to abiotic stress [48], [49].

Plants produce various endogenous signaling molecules, such as ABA, ethylene and H_2_O_2_, when subject to abiotic stress. Exogenous application of ABA, an established mediator of water stress response, enhanced root hydraulic conductivity in sunflower and maize [20], [21], and affected expression of *AQPs* in rice, *Craterostigma plantagineum*, Arabidopsis, and tobacco [50]–[53]. The response of each *AQP* to ABA is different, implying that the regulation of *AQP* expression involves both ABA-dependent and ABA-independent signaling pathways [54]. In addition, although several isoforms of AQPs are able to facilitate H_2_O_2_ transport across the tonoplast and plasma membrane [19], [22], H_2_O_2_ has been reported as an inhibitor of water transport by AQPs [17], [23], [55]–[57]. In wheat, the signaling pathway involved in regulating *AQPs* expression is poorly understood [14]. In the present study, the tungstate which is an inhibitor of ABA biosynthesis could inhibit the upregulation of *TaAQP7* under PEG treatment, suggesting that endogenous ABA signaling might be involved in the upregulation of *TaAQP7* induced by PEG treatment (Fig. 3A). However, DMTU which is a scavenger of H_2_O_2_, had no effect on the upregulation of *TaAQP7* after PEG and ABA treatment (Fig. 3B), implying that endogenous H_2_O_2_ might not contribute to the upregulation of *TaAQP7* induced by PEG and ABA treatments. These results indicate that although both exogenous ABA and H_2_O_2_ upregulated *TaAQP7*, they may play different roles in regulating *TaAQP7* expression after PEG treatment. In addition, the response of *TaAQP7* to MeJA and SA implys that *TaAQP7* maybe also involved in these molecules mediated signaling pathway.

In order to further understand the function of *TaAQP7* under drought stress, we generated transgenic tobacco plants overexpressing *TaAQP7* under the control of the constitutive CaMV 35S promoter. Transgenic plants exhibited improved tolerance against drought stress when compared to WT in one-, three- and six-week- old tobacco plants (Fig. 4 and 5). These results are consistent with previous studies on *AQP* genes conferring abiotic stress to plants [2], [4], [9], [11], [13], [14], [58], [59]. However, studies have also shown that overexpression of some AQP isoforms increased sensitivity to drought stress [11], [46], [60]. The various phenotypes of *AQPs*-overexpressing plants displayed under abiotic stress may be due to a cumulative effect on water transport property, transpiration rate and stomatal aperture [2], [4], [11], [25], [58].

Land plants have evolved to cope with rapid changes in the availability of water by regulating all aquaporins that play an important role in water uptake and movement [61], [62]. Numerous studies have shown that overexpression of *AQPs* enhanced the ability of plants to tolerate abiotic stress by improving water use efficiency, hydraulic conductivity and retaining better water status [2], [4], [11], [58], [59]. In our study, the RWC was similar in transgenic lines and WT under normal conditions, while under drought stress it was remarkably lower in WT when compared to the transgenic lines (Fig. 6A), implying that transgenic lines retain better water status than WT under drought stress. In addition, it is noteworthy to state that overexpression of *HvPIP2;1* that shares high similarity with *TaAQP7* also leads to an increased transpiration rate and slightly decreased intrinsic water-use efficiency under normal conditions [63]. This difference could be due to different conditions to which the tested plants were subjected. Thus, it is speculated that water loss stimulates the expression of *TaAQP7* to maintain better water status, supported by the upregulation of *TaAQP7* under dehydration and PEG treatments. Drought stress always induces a rapid accumulation of ROS and damages the cell membrane by oxidizing proteins, lipids, and DNA [64], [65]. Overexpression of *AQPs* was reported to decrease IL and MDA that are important indicators of membrane injury under stress. IL was found to be lower in *OsPIP2;7*-overexpressing rice when compared to the controls under chilling stress [59] and *BjPIP1*-overexpressing plants showed lower levels of IL and MDA under Cd stress [66]. IL and MDA measurements in the present study showed that transgenic lines had less IL and MDA than WT under drought stress (Fig. 6B and C) suggesting that transgenic lines were subjected to less membrane injury. MDA, the product of lipid peroxidation caused by ROS, is in general used to evaluate ROS-mediated injuries in plants [41]. Thus, these studies imply that the lipid peroxidation caused by ROS damage is relatively relieved in transgenic plants under drought stress.

ROS have been shown to alter aquaporin structure, thereby leading to channel closure through direct oxidative mechanism [67], or induce internalization of PIPs and reduce hydraulic conductivity through cell signaling mechanisms [23]. H_2_O_2_-mediated decrease in water transport under abiotic stress has been reported in previous studies. In the roots of Arabidopsis plants, NaCl concomitantly induced the accumulation of ROS and inhibition of hydraulic conductivity concomitantly [23]. Rapid accumulation of H_2_O_2_ in cucumber roots after exposure to low temperature resulted in a decrease in water transport [55]. The activities of AQPs were higher in chilling-tolerant genotype than in chilling-sensitive genotype due to less IL and H_2_O_2_ content in chilling-tolerant genotype during chilling stress [17]. In addition, the positive role of H_2_O_2_ in stress tolerance was also identified. Active BR signaling can induce gene expression of the nicotinamide adenine dinucleotide phosphate (NADPH) oxidase to regulate H_2_O_2_ production to confer abiotic and biotic stress tolerance [68]. Low concentration of H_2_O_2_ can increase the PIP protein abundance and promote the root growth in *Phaseolus vulgaris*, which reflects the dual function of ROS in plant [69]. However, whether overexpression of *AQPs* contributes to reduced ROS accumulation is uncertain. Therefore, the ROS level was measured in transgenic plants and WT in the present study. The results showed that transgenic lines accumulated much lower levels of H_2_O_2_ relative to WT in one- and three-week-old tobacco plants under drought/osmotic stress (Fig. 7A, 8A and B), which suggested that overexpression of *TaAQP7* was benefited for reducing H_2_O_2_ accumulation under drought stress.

In order to detoxify stress-induced ROS, plants have evolved a complex antioxidant system, in which several enzymes play essential roles such as scavenging ROS and protecting cells against oxidative stress [70]–[72]. In the present study, activities of antioxidant enzymes such as SOD and CAT were enhanced in one- (Fig. 8C and D) and three-week-old transgenic tobacco plants (Fig. 7B and C) under drought/osmotic stress, indicating that overexpression of *TaAQP7* effectively improved the antioxidant defense system, which in turn protected transgenic lines against ROS-mediated injury under drought/osmotic stress. Similarly, *BjPIP1*-overexpressing plants showed higher activities of antioxidant enzymes under Cd stress than WT [66], implying that antioxidant system may be involved in *AQPs* conferring abiotic stress in plants. Here, we have provided physiological evidence that *TaAQP7* confers drought stress tolerance by enhancing SOD and CAT activities. In addition, the transgenic lines had higher activities of SOD and CAT, and lower membrane injury than the WT when they were in the same water status under drought stress (Fig. 9). Therefore, higher antioxidant enzyme activity displayed by the transgenic plants under drought stress may not be due to the better water status but most likely due to less damage caused to the cell membrane which could protect the protein machinery from dehydration damage.

In conclusion, a *PIP2* subgroup *AQP* gene, *TaAQP7*, was cloned and characterized. *TaAQP7* confers drought stress tolerance not only by retaining better water status but also by reducing ROS accumulation and membrane damage via enhancing the activities and expression of antioxidant enzymes. Further work will emphasize the mechanism of *AQPs* on the sophisticated antioxidative system under drought stress.

## Materials and Methods

### Plant Materials and Treatments

Wheat (*Triticum aestivum* L. cv. Chinese Spring) seeds were surface-sterilized with 75% (v/v) ethanol for 2 min and 1% (v/v) bichloride for 10 min, and then washed with sterile water three times. The sterilized seeds germinated on sterile water and grown in Murashige and Skoog (MS) liquid medium under a 16 h light/8 h dark cycle at 25°C. For PEG treatment, the 10-day-old seedlings were transferred into Petri dishes containing 20% PEG6000 solution and the plants were incubated under light for different time. For signaling molecules treatments, the 10-day-old seedlings were sprayed with 100 µM ABA, 100 µM MeJA, 50 µM auxin, 2 mM salicylic acid or 10 mM H_2_O_2_ respectively and the plants were incubated under light for different time. For inhibitors treatment, the plants were pretreated with 1 mM tungstate, and 5 mM DMTU for 2 h and 6 h respectively, and then exposed to 20% PEG6000 for 2 h and 6 h respectively. The plants were pretreated with 5 mM DMTU for 2 h and 6 h respectively, and then exposed to 100 µM ABA for 2 h and 6 h respectively. The treatment of tungstate or DMTU alone was also performed in the experiement. In order to get reliable results, for all of the above treatments, the wheat seedlings of consistent growth were subjected to various treatments and the un-treated wheat seedlings were used as control for each series of treatments. For the organs differential expression assays, roots, stems, and leaves were cut from sterile seedlings, and pistils, stamens and lemma were obtained from wheat plants in the field. The samples from treated or control plants were subsequently frozen in liquid nitrogen and stored at −80°C for extraction of total RNA and qRT-PCR assay.

### Cloning and Bioinformatics Analysis of *TaAQP7* Gene

The wheat expressed sequence tags (ESTs) are available on the DFCI wheat gene index database (http://compbio.dfci.harvard.edu/cgi-bin/tgi/gimain.pl?gudb=wheat), from which an EST sequence (BE518349) belonging to major intrinsic protein (MIP) family was acquired. Sequence analysis by ORF Finder showed that the 3′-end was missing. Employing RACE reaction, the 3′- end of the gene from wheat was amplified with the SMART RACE cDNA amplification kit (Clontech) using the primer (P1, Table S1) and the cDNA obtained from leaves of wheat seedlings treated with 200 mM NaCl, 20% PEG6000, and cold (4°C) for 2 h as template. The amplified products of the 3′ cDNA ends were inserted into the pMD18-T vector (TaKaRa). Nucleotide sequences of the inserted cDNA fragment was determined on an ABI PRISM310 Genetic Analyzer (Perkin Elmer Applied Biosystems, Foster City, CA, USA) using the BigDye Termination Cycle Sequencing Ready Reaction Kit (Perkin Elmer). The full-length cDNA sequence was identified with the help of DNAMAN software and was amplified by reverse transcriptase-polymerase chain reaction (RT-PCR) with the primer (P2, Table S1) and wheat Poly (A)^+^ mRNA as template. The amplified product was inserted into the pMD18-T vector (TaKaRa). The nucleotide sequence was determined on an ABI PRISM310 Genetic Analyzer (Perkin Elmer) using the BigDye Termination Cycle Sequencing Ready Reaction Kit (Perkin Elmer). The sequence was analyzed by BLAST (http://ncbi.nlm.nih.gov/blast).

### qRT-PCR Analysis

The expression of *TaAQP7* in different wheat organs including leaves after different treatments was analyzed by qRT-PCR using the fluorescent intercalating dye SYBRGreen (ToYoBo, Japan) in a detection system (MJ research opticon2). Each 200 ng Poly(A)^+^ mRNA was converted into cDNA using AMV Reverse Transcriptase (Promega) at 42°C in a final volume of 20 µl, which subsequently served as the template in qRT-PCR. The primers (P3–P7, Table S1) used in the qRT-PCR excluded the highly conserved protein domain and had high efficiency and specificity, which was detected by Opticon monitor2 qRT-PCR software and agarose gel electrophoresis. The resulted PCR products by all the primers were subjected to sequence to confirm the specificity. In all of the experiments, appropriate negative controls containing no template RNA were subjected to the same procedure to exclude or detect any possible contamination. Before proceeding with the actual experiments, a series of template and primer dilutions were tested to determine the optimal template and primer concentration for maximum amplification of the target during the experiments. The amplification efficiencies for the internal control and the target genes were determined with the range from 0.9 to 1.1. Each sample was amplified in four independent replicates, and the data were analyzed using Opticon monitor 2 qRT-PCR software. *TaActin* or *NtUbiquitin* was used as the internal control for wheat and tobacco, respectively, that served as a benchmark to which other genes examined was normalized. The mRNA fold difference was relative to that of un-treated samples used as calibrator. The relative expression level of genes calculated using the 2^–ΔΔCt^ formula [73].

### Subcellular Localization of TaAQP7 Protein

The coding sequence of *TaAQP7*containing an *Nco*I/*Spe*I restriction site was amplified by using primers (P8, Table S1) for transient expression in onion epidermal cells. The PCR products were subcloned into *Nco*I/*Spe*I sites of pCAMBIA1304-GFP expression vector under control of the CMV 35S promoter. The pCAMBIA1304-TaAQP7-GFP and the pm-rk were transiently expressed in onion epidermal cells using gene gun (PDS-1000, BIO-RAD), where pm-rk was used as the plasma membrane-localized maker [43]. Fluorescence was observed by confocal laser scanning microscopy (LSM700; Carl Zeiss) after incubation at 25°C for 24 h on MS medium. For the subcellular localization of TaAQP7 in transgenic lines, root samples were sliced with razor blades and mounted between slides and cover slip in water. The ﬂuorescence of the GFP fusion proteins was observed with confocal laser scanning microscopy.

### Complementary RNA Synthesis, Oocyte Preparation, cRNA Injection, and Osmotic Water Permeability Assay

The cDNA of *TaAQP7* were subcloned into pCS107 vector using the ﬂanking restriction sites *BamH*I and *EcoR*I. The cRNA transcripts were synthesized *in vitro* with mMESSAGE mMACHINE High Yield Capped RNA Transcription Kit (Ambion) with *Asc*I linearized vector. Oocyte preparation, injection and expression were performed as described by Daniels et al. (1996) [74]. The *Xenopus laevis* oocytes of stages VI were isolated and stored in ND96 solution (96 mM NaCl, 2 mM KCl, 1 mM CaCl_2_, 1 mM MgCl_2_ and 5 mM Hepes, pH 7.4) containing 10 µg/ml streptomycin. The follicular cell layer was removed via 2 h of incubation with 2 mg/ml of collagenase in ND96 buffer at 25°C with continuous gentle agitation. The defolliculated oocytes were injected with 50 nl of cRNA (1 mg/ml) or water using as negative control and then the oocytes were incubated at 18°C for 48 h in ND96 solution supplemented with 10 µg/ml penicillin and 10 µg/ml streptomycin. Osmotic water permeability coefficient of oocytes was determined as described by Zhang and Verkman (1991) [75]. To measure the osmotic water permeability coefficient, oocytes were transferred to five-fold diluted ND96 solution. Changes in the oocytes volume were monitored at room temperature with a microscope video system by taking digital images at 30s intervals. Oocytes volumes (V) were calculated from the measured area of each oocyte. The osmotic Pf was calculated for the first 5 min using the formula Pf = V_0_ [d(V/V_0_)/dt]/[S_0_×*V_W_* (Osmin - Osmout)]. V_0_ and S_0_ are the initial volume and surface area of each individual oocyte, respectively; d(V/V0)/dt, the relative volume increase per unit time; *Vw*, the molar volume of water (18 cm^3^ mol^−1^); and Osmin - Osmout, the osmotic gradient between the inside and outside of the oocyte.

### Plant Transformation and Generation of Transgenic Plants

The recombinant plasmids pCAMBIA1304-TaAQP7-GFP was introduced into the *Agrobacterium tumefaciens* strain LBA4404. The transgenic tobaccos were generated using *Agrobacterium*-mediated transformation method [76]. The seeds from overexpression transgenic plants were selected on MS medium containing 40 mg/L of hygromycin. The hygromycin-resistant T_1_ seedlings were confirmed by RT-PCR amplification using the primers for *TaAQP7* (P9, Table S1) and *GFP* (P10, Table S1) gene. Three independent transgenic T_2_ line seedlings that almost all survived on MS medium containing 40 mg/L of hygromycin were used in the experiments. The expression of *TaAQP7* in the three independent T_2_ lines was investigated by semi-quantitative RT-PCR analysis using primers P9 and P5 (Table S1), among which the *NtUbiquitin* gene was used as an internal control.

### Stress Tolerance Assays of the WT and the Transgenic Plants

WT, VC and transgenic lines were cultured in MS medium under a 16 h light/8 h dark cycle at 25°C for one week and then transplanted to containers filled with a mixture of soil and sand (3∶1) where they were regularly watered for two weeks or five weeks for the drought stress tolerance assay. Plants with consistent growth state were subjected to withhold water for 20 d. After 20 d withholding water, photographs were taken. In the drought stress treatment, the leaves were collected from three-week-old plants to measure RWC, IL, MDA, H_2_O_2_, SOD and CAT at 15 and 30 d drought treatment respectively. In the mannitol treatment, the WT and transgenic lines were cultured in MS medium under a 16 h light/8 h dark cycle at 25°C for one week, and then the seedlings were transferred to MS or MS supplied with 150 or 300 mM mannitol for one week. The whole seedlings were used to perform physiological experiments, in which the root length, H_2_O_2_ content, SOD and CAT activities were measured. A total of 200 surface-sterilized seeds of each transgenic line, VC or WT were sowed on MS without mannitol or MS supplied with 300 mM mannitol for 12 d to detect the germination rate.

### Measurement of RWC, MDA Content and IL

For the RWC assay in wheat, RWC was measured in control and dehydration-stressed 10-day-old seedlings. For dehydration stress, 10-day-old seedlings were placed onto dry filter paper within 12 h and the fresh weight (FW) of wheat leaves was immediately recorded. The leaves were soaked for 4 h in distilled water at room temperature under a constant light, and the turgid weight (TW) was recorded. After drying for 24 h at 80°C total dry weight (DW) was recorded. For the RWC assay in tobacco, three-week-old tobacco plants were deprived of water for 30 d. Tobacco leaves were sampled from WT and transgenic lines under drought stress for 15 d and 30 d to detect RWC. Fresh weight (FW) of plants was immediately recorded after leaf excision. The plants were soaked for 4 h in distilled water at room temperature under a constant light, and the turgid weight (TW) was recorded. After drying for 24 h at 80°C total dry weight (DW) was recorded. RWC was calculated as follows: RWC (%) = [(FW − DW)/(TW −DW)]×100 [77]. MDA content was determined by the thiobarbituric acid (TBA)-based colorimetric method as described by Heath and Packer [78]. IL was measured based on the method of Jiang and Zhang [35] with slight modification. The collected leaves were cut into strips and incubated in 10 ml of distilled water at room temperature for 12 h. The initial conductivity (C1) was measured with a conductivity meter (DDBJ-350, Shanghai, China). The samples were then boiled for 30 min to result in complete ion leakage. After cooling down at room temperature, the electrolyte conductivity (C2) was measured. Electrolyte leakage (C) was calculated according to the equation C (%) = C1/C2×100.

### Measurement of SOD,CAT and POD Activities and H_2_O_2_ Content

The activities of three antioxidant enzymes, CAT, POD, and SOD were measured by spectrophotometer. In the SOD, POD and CAT activities measurement, each sample (0.5 g) was ground in liquid and homogenized in 5 ml of extraction buffer containing 0.05 M phosphate buffer (pH 7.8) and 1% polyvinylpyrrolidone. The homogenate was centrifuged at 10000 g for 10 min at 4°C and the resulting supernatant was collected for enzyme activity analysis. SOD and CAT activities were spectrophotometrically measured by using SOD and CAT Detection Kit (A001 and A007, Jiancheng, Nanjing, China) according to the manufacturer’s instruction. Total POD activity was measured by the change in absorbance of 470 nm due to guaiacol oxidation according to the method described in previous study [79]. Samples were collected for H_2_O_2_ measurements as described in previous study [35].

### Histochemical Detection of H_2_O_2_

H_2_O_2_ accumulation was detected by the DAB staining methods [80]. The seedlings infiltrated with 5 mg/ml DAB at pH 3.8 for 20 h to detect H_2_O_2_. Then the seedlings were decolorized by boiling in ethanol (96%) for 10 min. After cooling, the seedlings were extracted at room temperature with fresh ethanol and photographed.

## Supporting Information

Figure S1 **Comparison of TaAQP7 with other known PIP proteins.** Amino acid sequences are aligned by ClusterX software. Six transmembrane-helix (H1–H6) are shown in the box. Letters marked with double transverse lines refer to the most highly conserved amino acid sequences of MIP. Letters marked with black dot represent the ‘NPA’ motif. The accession numbers of these known proteins in GenBank are as follows: HvPIP2;1 (BAA23744.1), ZmPIP2;2 (ACG33001.1), ZmPIP1;1 (AAO86706.1) and HvPIP1;1 (BAF41978.1). The accession numbers of these known proteins in GenBank are given in parentheses.
(TIF)

Figure S2 **Phylogenetic relationships of TaAQP7 (boxed) with other known AQPs.** The unrooted tree was constructed using full-length amino acid sequences and summarized the phylogenetic relationship among the members of AQP family in wheat, Arabidopsis and rice. Tree was made using ClustalX 1.83 and MEGA 4.0.
(TIF)

Figure S3 **Expression analysis of** ***TaAQP7*** **in different organs in wheat by qRT-PCR.** R: root; S: stem; L: leaf; ST: stamen; P: pistil; LE: lemma. The y-axis represents the relative fold difference in mRNA level calculated using the 2^–ΔΔCt^ formula with *TaActin* as internal control. The mRNA fold difference was relative to that of leaf samples used as calibrator. Vertical bars indicate ±SE of four replicates on one sample. When no bar is shown, the deviation is smaller than the symbol. Three biological experiments were performed, which produced similar results.
(TIF)

Figure S4 **Expression profiles of** ***TaAQP7*** **under MeJA, SA and auxin treatments in wheat.** Ten-day-old wheat seedlings were treated with 100 µM MeJA (A), 2 mM SA (B), and 50 µM auxin (C) and leaves were sampled within 24 h to extract RNA for qRT-PCR analysis. The y-axis represents the relative fold difference in mRNA level calculated using the 2^–ΔΔCt^ formula with *TaActin* as internal control. The mRNA fold difference is relative to that of distilled water treated samples used as calibrator. Vertical bars indicate ±SE of four replicates on one sample. When no bar is shown, the deviation is smaller than the symbol. Three biological experiments were performed, which produced similar results.
(TIF)

Video S1 **The oocytes swelling in** ***TaAQP7*** **-expressing oocytes.** The defolliculated oocytes were injected with 50 nl of cRNA (1 mg/ml) or water using as negative control and then the oocytes were incubated at 18°C for 48 h in ND96 solution supplemented with 10 µg/ml penicillin and 10 µg/ml streptomycin. The oocytes were transferred to five-fold diluted ND96 solution, and then the oocytes volume were monitored at room temperature with a microscope video system by taking digital images at 30s intervals. The upper (yellow) is the control and the lower (blue) is the oocytes injected with cRNA of *TaAQP7*. Three biological experiments were performed, which produced similar results.
(MOV)

Table S1 **Primers used for PCR analysis.**
(DOC)

## References

[1] Suga S, Komatsu S, Maeshima M (2002) Aquaporin isoforms responsive to salt and water stresses and phytohormones in radish seedlings. Plant Cell Physiol 43: 1229–1237. 12407203 10.1093/pcp/pcf148

[2] Lian HL, Xin Y, Ye Q, Ding XD, Kitagawa Y, et al (2004) The role of aquaporin RWC3 in drought avoidance in rice. Plant Cell Physiol 45: 481–489. 15111723 10.1093/pcp/pch058

[3] Vera-Estrella R, Barkla BJ, Bohnert HJ, Pantoja O (2004) Novel regulation of aquaporins during osmotic stress. Plant Physiol 135: 2318–2329. 15299122 10.1104/pp.104.044891 PMC520800

[4] Sade N, Gebretsadik M, Seligmann R, Schwartz A, Wallach R, et al (2010) The role of tobacco Aquaporin1 in improving water use efficiency, hydraulic conductivity, and yield production under salt stress. Plant Physiol 152: 245–254. 19939947 10.1104/pp.109.145854 PMC2799360

[5] Johanson I, Gustavsson S, Jovall S, Fraysse F (2001) The complete set of genes encoding major intrinsic proteins in Arabidopsis provides a framework for a new nomenclature for major intrinsic proteins in plants. Plant Physiol 126: 1358–1369. 11500536 10.1104/pp.126.4.1358 PMC117137

[6] Chaumont F, Barrieu F, Wojcik E, Chrispeels MJ, Jung R (2001) Aquaporins constitute a large and highly divergent protein family in maize. Plant Physiol 12: 1206–1215. 10.1104/pp.125.3.1206 PMC65601 11244102

[7] Sakurai J, Ishikawa F, Yamaguchi T, Uemura M, Maeshima M (2005) Identification of 33 rice aquaporin genes and analysis of their expression and function. Plant Cell Physiol 46: 1568–1577. 16033806 10.1093/pcp/pci172

[8] Aroca R, Amodeo G, Fernández-Illescas S, Herman EM, Chaumont F, et al (2004) The role of aquaporins and membrane damage in chilling and hydrogen peroxide induced changes in the hydraulic conductance of maize roots. Plant Physiol 137: 341–353. 15591439 10.1104/pp.104.051045 PMC548864

[9] Guo L, Wang ZY, Lin H, Cui WE, Chen J, et al (2006) Expression and functional analysis of the rice plasma-membrane intrinsic protein gene family. Cell Res 16: 277–286. 16541126 10.1038/sj.cr.7310035

[10] Yu X, Peng YH, Zhang MH, Shao YJ, Su WA, et al (2006) Water relations and an expression analysis of plasma membrane intrinsic proteins in sensitive and tolerant rice during chilling and recovery. Cell Res 16: 599–608. 16775631 10.1038/sj.cr.7310077

[11] Cui XH, Hao FS, Chen H, Chen J, Wang XC (2008) Expression of the *Vicia faba VfPIP1* gene in *Arabidopsis thaliana* plants improves their drought resistance. J Plant Res 121: 207–214. 18193401 10.1007/s10265-007-0130-z

[12] Mahdieh M, Mostajeran A, Horie T, Katsuhara M (2008) Drought stress alters water relations and expression of PIP-type aquaporin genes in *Nicotiana tabacum* plants. Plant Cell Physiol 49: 801–813. 18385163 10.1093/pcp/pcn054

[13] Peng YH, Arora R, Li GW, Wang X, Fessehaie A (2008) Rhododendron catawbiense plasma membrane intrinsic proteins are aquaporins and their overexpression compromises constitutive freezing tolerance and cold acclimation ability of transgenic Arabidopsis plants. Plant Cell Environ 31: 1275–1289. 18518915 10.1111/j.1365-3040.2008.01840.x

[14] Gao ZX, He XL, Zhao BC, Zhou CJ, Liang YZ, et al (2010) Overexpressing a putative aquaporin gene from wheat, *TaNIP*, enhances salt tolerance in transgenic Arabidopsis. Plant Cell Physiol 51: 767–775. 20360019 10.1093/pcp/pcq036

[15] Johansson I, Karlsson M, Johanson U, Larsson C, Kjellbom P (2000) The role of aquaporins in cellular and whole plant water balance. Biochim Biophys Acta 1465: 324–342. 10748263 10.1016/s0005-2736(00)00147-4

[16] North GB, Nobel PS (2000) Heterogeneity in water availability alters cellular development and hydraulic conductivity along roots of a desert succulent. Ann Bot 85: 247–255.

[17] Aroca R, Amodeo G, Fernández-Illescas S, Herman EM, et al (2005) The Role of Aquaporins and Membrane Damage in Chilling and Hydrogen Peroxide Induced Changes in the Hydraulic Conductance of Maize Roots. Plant Physiol 137: 341–353. 15591439 10.1104/pp.104.051045 PMC548864

[18] Horie T, Kaneko T, Sugimoto G, Sasano S, Panda SK, et al (2011) Mechanisms of water transport mediated by PIP aquaporins and their regulation via phosphorylation events under salinity stress in barley roots. Plant Cell Physiol 52: 663–675. 21441236 10.1093/pcp/pcr027

[19] Bienert GP, Schjoerring JK, Jahn TP (2006) Membrane transport of hydrogen peroxide. Biochim Biophys Acta 1758: 994–1003. 16566894 10.1016/j.bbamem.2006.02.015

[20] Quintero JM, Fournier JM, Benlloch M (1999) Water transport in sunﬂower root systems: effects of ABA, Ca^2+^ status and HgCl_2_ . J Exp Bot 50: 1607–1612.

[21] Hose E, Steudle E, Hartung W (2000) Abscisic acid and hydraulic conductivity of maize roots: a study using cell- and root-pressure probes. Planta 211: 874–882. 11144273 10.1007/s004250000412

[22] Dynowski M, Schaaf G, Loque D, Moran O, Ludewig U (2008) Plant plasma membrane water channels conduct the signaling molecule H_2_O_2_ . Biochem J 414: 53–61. 18462192 10.1042/BJ20080287

[23] Boursiac Y, Boudet J, Postaire O, Luu DT, Tournaire-Roux C, et al (2008) Stimulus-induced downregulation of root water transport involves reactive oxygen species-activated cell signalling and plasma membrane intrinsic protein internalization. Plant J 56: 207–218. 18573191 10.1111/j.1365-313X.2008.03594.x

[24] Boursiac Y, Prak S, Boudet J, Postaire O, Luu DT, et al (2008) The response of Arabidopsis root water transport to a challenging environment implicates reactive oxygen species- and phosphorylation-dependent internalization of aquaporins. Plant Signal Behav 3: 1096–1098. 19704504 10.4161/psb.3.12.7002 PMC2634465

[25] Heinen RB, Ye Q, Chaumont F (2009) Role of aquaporins in leaf physiology. J Exp Bot 60: 2971–2985. 19542196 10.1093/jxb/erp171

[26] Ehlert C, Maurel C, Tardieu F, Simonneau T (2009) Aquaporin-mediated reduction in maize root hydraulic conductivity impacts cell turgor and leaf elongation even without changing transpiration. Plant Physiol 150: 1093–1104. 19369594 10.1104/pp.108.131458 PMC2689965

[27] Li DD, Wu YJ, Ruan XM, Li B, Zhu L, et al (2009) Expressions of three cotton genes encoding the PIP proteins are regulated in root development and in response to stresses. Plant Cell Rep 28: 291–300. 18956193 10.1007/s00299-008-0626-6

[28] Flower DJ, Ludlow MM (1986) Contribution of osmotic adjustment to the dehydration tolerance of water-stressed pigeon pea (*Cajanus cajan* (L.) *millsp*.) leaves. Plant Cell Environ 9: 33–40.

[29] Rampino P, Pataleo S, Gerardi C, Mita G, Perrotta C (2006) Drought stress response in wheat: physiological and molecular analysis of resistant and sensitive genotypes. Plant Cell Environ 29: 2143–2152. 17081248 10.1111/j.1365-3040.2006.01588.x

[30] Jiang MY, Zhang JH (2002) Water stress-induced abscisic acid accumulation triggers the increased generation of reactive oxygen species and up-regulates the activities of antioxidant enzymes in maize leaves. J Exp Bot 53: 2401–2410. 12432032 10.1093/jxb/erf090

[31] Zhang AY, Jiang MY, Zhang JH, Tan MP, Hu XL (2006) Mitogen activated protein kinase is involved in abscisic acid-induced antioxidant defense and acts downstream of reactive oxygen species production in leaves of maize plants. Plant Physiol 141: 475–487. 16531486 10.1104/pp.105.075416 PMC1475456

[32] Mittler R, Herr EH, Orvar BL, van Camp W, Willekens H, et al (1999) Transgenic tobacco plants with reduced capability to detoxify reactive oxygen intermediates are hyperresponsive to pathogen infection. Proc Natl Acad Sci U S A 96: 14165–14170. 10570216 10.1073/pnas.96.24.14165 PMC24208

[33] Guan LM, Zhao J, Scandalios JG (2000) Cis-elements and trans-factors that regulate expression of the maize Cat1 antioxidant gene in response to ABA and osmotic stress: H_2_O_2_ is the likely intermediary signaling molecule for the response. Plant J 22: 87–95. 10792824 10.1046/j.1365-313x.2000.00723.x

[34] Pei ZM, Murata Y, Benning G, Thomine S, Klüsener B, et al (2000) Calcium channels activated by hydrogen peroxide mediate abscisic acid signaling in guard cells. Nature 406: 731–734. 10963598 10.1038/35021067

[35] Jiang M, Zhang J (2001) Effect of abscisic acid on active oxygen species, antioxidative defence system and oxidative damage in leaves of maize seedlings. Plant Cell Physiol 42: 1265–1273. 11726712 10.1093/pcp/pce162

[36] Lin CC, Kao CH (2001) Abscisic acid induced changes in cell wall peroxidase activity and hydrogen peroxide level in roots of rice seedlings. Plant Sci 160: 323–329. 11164604 10.1016/s0168-9452(00)00396-4

[37] Kwak JM, Mori IC, Pei ZM, Leonhardt N, Torres MA, et al (2003) NADPH oxidase *AtrbohD and AtrbohF* genes function in ROS-dependent ABA signaling in Arabidopsis. EMBO J 22: 2623–2633. 12773379 10.1093/emboj/cdg277 PMC156772

[38] Kuo TH, Kao CH (2004) Hydrogen peroxide is necessary for abscisic acid-induced senescence of rice leaves. J Plant Physiol 161: 1347–1357. 15658805 10.1016/j.jplph.2004.05.011

[39] Laloi C, Mestres-Ortega D, Marco Y, Meyer Y, Reichheld JP (2004) The Arabidopsis cytosolic h5 gene induction by oxidative stress and its w-box-mediated response to pathogen elicitor. Plant Physiol 134: 1006–1016. 14976236 10.1104/pp.103.035782 PMC389923

[40] Hu X, Jiang M, Zhang A, Lu J (2005) Abscisic acid-induced apoplastic H_2_O_2_ accumulation up-regulates the activities of chloroplastic and cytosolic antioxidant enzymes in maize leaves. Planta 223: 57–68. 16049674 10.1007/s00425-005-0068-0

[41] Moore K, Roberts LJ (1998) Measurement of lipid peroxidation. Free Radic Res 28: 659–671. 9736317 10.3109/10715769809065821

[42] Hanson MR, Kohler RH (2001) GFP imaging: methodology and application to investigate cellular compartmentation in plants. J Exp Bot 356: 529–538. 11373302

[43] Nelson BK, Cai X, Nebenführ A (2007) A multicolored set of in vivo organelle markers for co-localization studies in Arabidopsis and other plants. Plant J 51: 1126–1136. 17666025 10.1111/j.1365-313X.2007.03212.x

[44] Forrest K, Bhave M (2008) The PIP and TIP aquaporins in wheat form a large and diverse family with unique gene structures and functionally important features. Funct Integr Genom 8: 115–133. 10.1007/s10142-007-0065-4 18030508

[45] Ayadi M, Cavez D, Miled N, Chaumont F, Masmoudi K (2011) Identification and characterization of two plasma membrane aquaporins in durum wheat (*Triticum turgidum* L. subsp. durum) and their role in abiotic stress tolerance. Plant Physiol Biochem 49: 1029–1039. 21723739 10.1016/j.plaphy.2011.06.002

[46] Jang JY, Kim DG, Kim YO, Kim JS, Kang H (2004) An expression analysis of a gene family encoding plasma membrane aquaporins in response to abiotic stresses in *Arabidopsis thaliana* . Plant Mol Biol 54: 713–725. 15356390 10.1023/B:PLAN.0000040900.61345.a6

[47] Alexandersson E, Fraysse L, Sjovall-Larsen S, Gustavsson S, Fellert M, et al (2005) Whole gene family expression and drought stress regulation of aquaporins. Plant Mol Biol 59: 469–484. 16235111 10.1007/s11103-005-0352-1

[48] Lian HL, Yu X, Lane D, Sun WN, Tang ZC, et al (2006) Upland rice and lowland rice exhibited different *PIP* expression under water deficit and ABA treatment. Cell Res 16: 651–660. 16773042 10.1038/sj.cr.7310068

[49] Wei H, Dhanaraj AL, Arora R, Rowland LJ, Fu Y, et al (2006) Identification of cold acclimation-responsive Rhododendron genes for lipid metabolism, membrane transport and lignin biosynthesis: importance of moderately abundant ESTs in genomic studies. Plant Cell Environ 29: 558–570. 17080607 10.1111/j.1365-3040.2005.01432.x

[50] Kaldenhoff R, Kölling A, Richter G (1993) A novel blue light- and abscisic acid-inducible gene of *Arabidopsis thaliana* encoding an intrinsic membrane protein. Plant Mol Biol 23: 1187–1198. 8292783 10.1007/BF00042352

[51] Weig A, Deswarte C, Chrispeels MJ (1997) The major intrinsic protein family of Arabidopsis has 23 members that form three distinct groups with functional aquaporins in each group. Plant Physiol 114: 1347–1357. 9276952 10.1104/pp.114.4.1347 PMC158427

[52] Mariaux JB, Bockel C, Salamini F, Bartels D (1998) Desiccation- and abscisic acid-responsive genes encoding major intrinsic proteins (MIPs) from the resurrection plant *Craterostigma plantagineum* . Plant Mol Biol 38: 1089–1099. 9869415 10.1023/a:1006013130681

[53] Siefritz F, Biela A, Eckert M, Otto B, Uehlein N, et al (2001) The tobacco plasma membrane aquaporin NtAQP1. J Exp Bot 52: 1953–1957. 10.1093/jexbot/52.363.1953 11559730

[54] Wan X, Steudle E, Hartung W (2004) Gating of water channels (aquaporins) in cortical cells of young corn roots by mechanical stimuli (pressure pulses): effects of ABA and of HgCl_2_ . J Exp Bot 55: 411–422. 14739264 10.1093/jxb/erh051

[55] Lee SH, Singh AP, Chung GC (2004) Rapid accumulation of hydrogen peroxide in cucumber roots due to exposure to low temperature appears to mediate decreases in water transport. J Exp Bot 55: 1733–1741. 15208334 10.1093/jxb/erh189

[56] Ye Q, Holbrook NM, Zwieniecki MA (2008) Cell-to-cell pathway dominates xylem-epidermis hydraulic connection in *Tradescantia ﬂuminensis* (Vell. Conc.) leaves. Planta 227: 1311–1319. 18273638 10.1007/s00425-008-0703-7

[57] Kim YX, Steudle E (2009) Gating of aquaporins by light and reactive oxygen species in leaf parenchyma cells of the midrib of *Zea mays* . J Exp Bot 60: 547–556. 19088335 10.1093/jxb/ern299 PMC2651454

[58] Zhang J, Deng Z, Cao S, Wang X, Zhang A, et al (2008) Isolation of six novel aquaporin genes from *Triticum aestivum* L. and functional analysis of *TaAQP6* in water redistribution. Plant Mol Biol Rep 26: 32–45.

[59] Li GW, Zhang MH, Cai WM, Sun WN, Su WA (2008) Characterization of OsPIP2;7, a water channel protein in rice. Plant Cell Physiol 49: 1851–1858. 18988636 10.1093/pcp/pcn166

[60] Aharon R, Shahak Y, Wininger S, Bendov R, Kapulnik Y, et al (2003) Overexpression of a plasma membrane aquaporin in transgenic tobacco improves plant vigor under favorable growth conditions but not under drought or salt stress. The Plant Cell 15: 439–447. 12566583 10.1105/tpc.009225 PMC141212

[61] Maurel C (1997) Aquaporins and water permeability of plant membranes. Annu Rev Plant Physiol Plant Mol Biol 48: 399–429. 15012269 10.1146/annurev.arplant.48.1.399

[62] Zhao CX, Shao HB, Chu LY (2008) Aquaporin structure-function relationships: water flow through plant living cells. Colloids Surf B Biointerfaces 62: 163–172. 18063350 10.1016/j.colsurfb.2007.10.015

[63] Hanba YT, Shibasaka M, Hayashi Y, Hayakawa T, Kasamo K, et al (2004) Overexpression of the barley aquaporin *HvPIP2;1* increases internal CO(2) conductance and CO(2) assimilation in the leaves of transgenic rice plants. Plant Cell Physiol 45: 521–529. 15169933 10.1093/pcp/pch070

[64] Polle A (2001) Dissection the superoxide dismutase-ascorbate-glutathione pathway by metabolic modeling: computer analysis as a step towards flux analysis. Plant Physiol 126: 445–462. 11351106 10.1104/pp.126.1.445 PMC102317

[65] Mittler R, Vanderauwera S, Gollery M, Van Breusegem F (2004) Reactive oxygen gene network of plants. Trends Plant Sci 9: 490–498. 15465684 10.1016/j.tplants.2004.08.009

[66] Zhang Y, Wang Z, Chai T, Wen Z, Zhang H (2008) Indian mustard aquaporin improves drought and heavy-metal resistance in tobacco. Mol Biotechnol 40: 280–292. 18622723 10.1007/s12033-008-9084-1

[67] Kourie JI (1998) Interaction of reactive oxygen species with ion transport mechanisms. Am J Physiol 275: C1–C24. 9688830 10.1152/ajpcell.1998.275.1.C1

[68] Choudhary SP, Yu JQ, Yamaguchi-Shinozaki K, Shinozaki K, Tran LS (2012) Benefits of brassinosteroid crosstalk. Trends Plant Sci 17: 594–60. 22738940 10.1016/j.tplants.2012.05.012

[69] Benabdellah K, Ruiz-Lozano JM, Aroca R (2009) Hydrogen peroxide effects on root hydraulic properties and plasma membrane aquaporin regulation in *Phaseolusvulgaris* . Plant Mol Biol 70: 647–661. 19437122 10.1007/s11103-009-9497-7

[70] Miller G, Suzuki N, Ciftci-Yilmaz S, Mittler R (2010) Reactive oxygen species homeostasis and signalling during drought and salinity stresses. Plant Cell Environ 33: 453–467. 19712065 10.1111/j.1365-3040.2009.02041.x

[71] Jaleel CA, Riadh K, Gopi R, Manivannan P, Inès J, et al (2009) Antioxidant defense responses: physiological plasticity in higher plants under abiotic constraints. Acta Physiol Plant 31: 427–436.

[72] Huang XS, Liu JH, Chen XJ (2010) Overexpression of *PtrABF* gene, a bZIP transcription factor isolated from Poncirus trifoliata, enhances dehydration and drought tolerance in tobacco via scavenging ROS and modulating expression of stress-responsive genes. BMC Plant Biol 10: 230. 20973995 10.1186/1471-2229-10-230 PMC3017851

[73] Livak KJ, Schmittgen TD (2001) Analysis of relative gene expression data using real-time Quantitative PCR and the 2^–ΔΔCt^ method. Methods 25: 402–408. 11846609 10.1006/meth.2001.1262

[74] Daniels MJ, Chaumont F, Mirkov TE, Chrispeels MJ (1996) Characterization of a new vacuolar membrane aquaporin sensitive to mercury at a unique site. Plant Cell 8: 587–599. 8624437 10.1105/tpc.8.4.587 PMC161122

[75] Zhang RB, Verkman AS (1991) Water and urea permeability properties of *Xenopus* oocytes: expression of mRNA from toadurinary bladder. Am J Physiol 260: C26–C34. 1987778 10.1152/ajpcell.1991.260.1.C26

[76] Horsch RB, Fry JE, Hoffmann NL, Eichholtz D, Rogers SC, et al (1985) A simple and general method for transferring genes into plants. Science 227: 1229–1231. 17757866 10.1126/science.227.4691.1229

[77] Barrs HD, Weatherley PE (1962) A reexamination of the relative turgidity technique for estimating water deficit in leaves. Aust J Biol Sci 15: 413–428.

[78] Heath RL, Packer L (1968) Photoperoxidation in isolated chloroplasts. I. Kinetics and stoichiometry of fatty acid peroxidation. Arch Biochem Biophys 125: 189–198. 5655425 10.1016/0003-9861(68)90654-1

[79] Polle A, Otter T, Seifert F (1994) Apoplastic peroxidases and lignification in needles of norway spruce (*Picea abies* L.). Plant Physiol 106: 53–60. 12232302 10.1104/pp.106.1.53 PMC159498

[80] Pan J, Zhang M, Kong X, Xing X, Liu Y, et al (2012) *ZmMPK17*, a novel maize group D MAP kinase gene, is involved in multiple stress responses. Planta 235: 661–676. 22006107 10.1007/s00425-011-1510-0

