# Supplementary figures and images for: Overexpression of the Wheat Aquaporin Gene, TaAQP7, Enhances Drought Tolerance in Transgenic Tobacco
Source: PLoS One. 2012 Dec 20;7(12):e52439. doi: 10.1371/journal.pone.0052439 (PMC3527513; doi:10.1371/journal.pone.0052439)

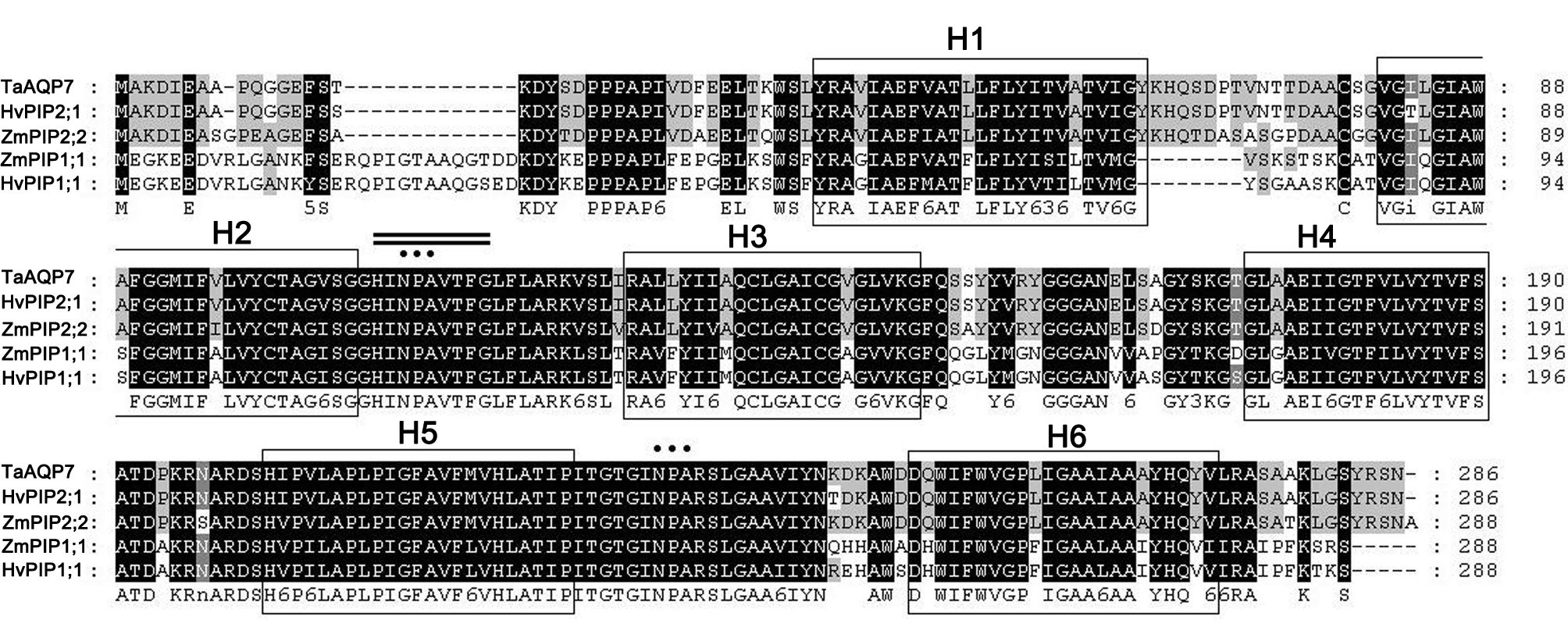

Supplement: Figure S1 — Comparison of TaAQP7 with other known PIP proteins. Amino acid sequences are aligned by ClusterX software. Six transmembrane-helix (H1–H6) are shown in the box. Letters marked with double transverse lines refer to the most highly conserved amino acid sequences of MIP. Letters marked with black dot represent the ‘NPA’ motif. The accession numbers of these known proteins in GenBank are as follows: HvPIP2;1 (BAA23744.1), ZmPIP2;2 (ACG33001.1), ZmPIP1;1 (AAO86706.1) and HvPIP1;1 (BAF41978.1). The accession numbers of these known proteins in GenBank are given in parentheses. (TIF) [file pone.0052439.s001.tif]

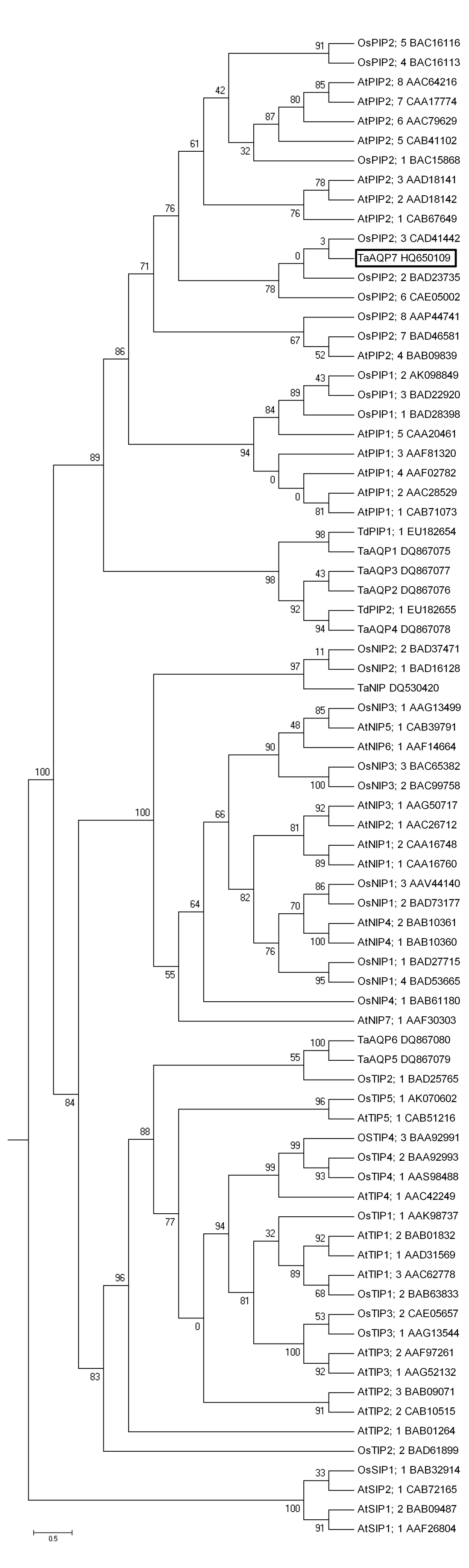

Supplement: Figure S2 — Phylogenetic relationships of TaAQP7 (boxed) with other known AQPs. The unrooted tree was constructed using full-length amino acid sequences and summarized the phylogenetic relationship among the members of AQP family in wheat, Arabidopsis and rice. Tree was made using ClustalX 1.83 and MEGA 4.0. (TIF) [file pone.0052439.s002.tif]

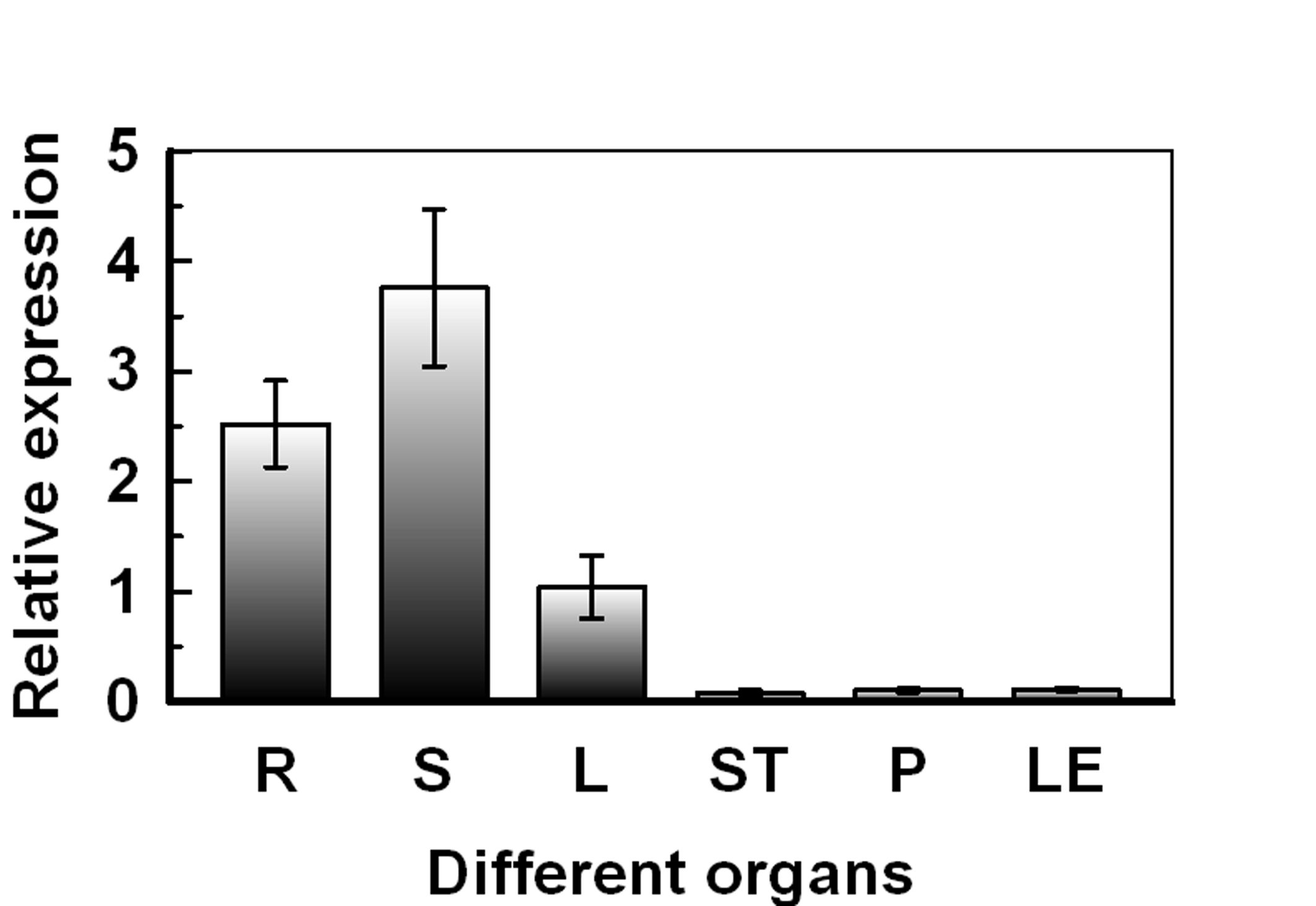

Supplement: Figure S3 — Expression analysis of TaAQP7 in different organs in wheat by qRT-PCR. R: root; S: stem; L: leaf; ST: stamen; P: pistil; LE: lemma. The y-axis represents the relative fold difference in mRNA level calculated using the 2–ΔΔCt formula with TaActin as internal control. The mRNA fold difference was relative to that of leaf samples used as calibrator. Vertical bars indicate ±SE of four replicates on one sample. When no bar is shown, the deviation is smaller than the symbol. Three biological experiments were performed, which produced similar results. (TIF) [file pone.0052439.s003.tif]

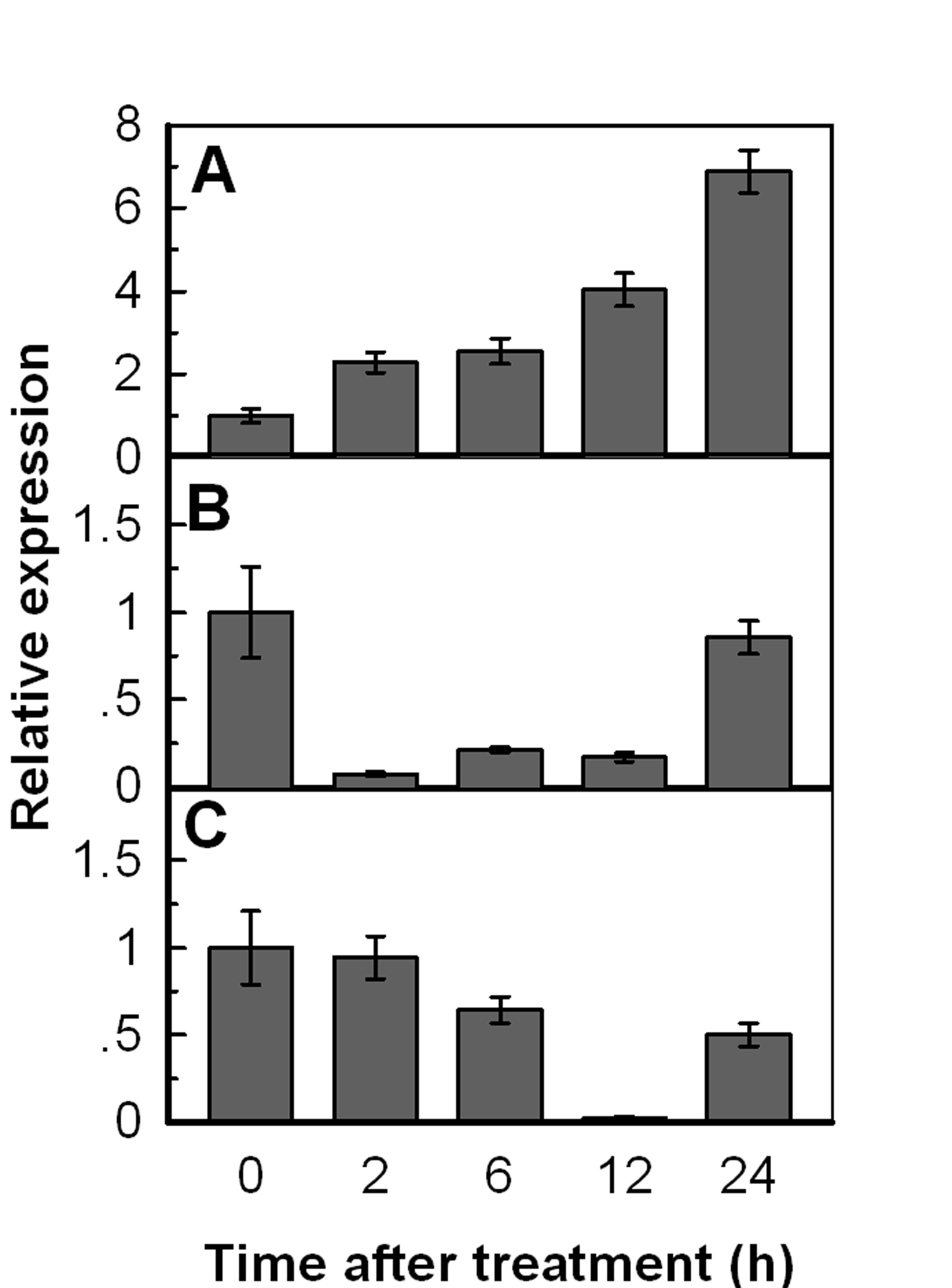

Supplement: Figure S4 — Expression profiles of TaAQP7 under MeJA, SA and auxin treatments in wheat. Ten-day-old wheat seedlings were treated with 100 µM MeJA (A), 2 mM SA (B), and 50 µM auxin (C) and leaves were sampled within 24 h to extract RNA for qRT-PCR analysis. The y-axis represents the relative fold difference in mRNA level calculated using the 2–ΔΔCt formula with TaActin as internal control. The mRNA fold difference is relative to that of distilled water treated samples used as calibrator. Vertical bars indicate ±SE of four replicates on one sample. When no bar is shown, the deviation is smaller than the symbol. Three biological experiments were performed, which produced similar results. (TIF) [file pone.0052439.s004.tif]
