# Supplementary material for: Overexpression of the Wheat Aquaporin Gene, TaAQP7, Enhances Drought Tolerance in Transgenic Tobacco
Source: PLoS One. 2012 Dec 20;7(12):e52439. doi: 10.1371/journal.pone.0052439 (PMC3527513; doi:10.1371/journal.pone.0052439)
Supplement: Table S1 — Primers used for PCR analysis. (DOC) [file pone.0052439.s006.doc]

**Table S1** Primers used for PCR analysis.

| Gene | Primer | Forward primer (5’-3’) | Reverse primer (5’-3’) |
| --- | --- | --- | --- |
| *TaAQP7* | P1 | CGGCGTCTCAGGTGGGCACATCAA | CTAATACGACTCACTATAGGGC |
| *TaAQP7* | P2 | CTAGCAATGGCGAAGGACAT | ACAGGACAAAGGTGTGGGAT |
| *TaAQP7* | P3 | GGCCGGACTGAAGTGTAGAT | ACAGGACAAAGGTGTGGGAT |
| *TaActin* | P4 | TGCTATCCTTCGTTTGGACCTT | AGCGGTTGTTGTGAGGGAGT |
| *NtUbiquitin* | P5 | TCCAGGACAAGGAGGGTAT | CATCAACAACAGGCAACCTAG |
| *NtSOD* | P6 | CTCCTACCGTCGCCAAAT | GCCCAACCAAGAGAACCC |
| *NtCAT* | P7 | AGGTACCGCTCATTCACACC | AAGCAAGCTTTTGACCCAGA |
| *TaAQP7* | P8 | CATGCCATGGCAATGGCGAAGGACATTGA | GGACTAGTGTTGCTCCTGTAGGACCCGA |
| *TaAQP7* | P9 | CTACACCGTCTTCTCCGC | ATCCATTGGTCATCCCAG |
| *mGFP5* | P10 | TGGAGAGGGTGAAGGTGA | CTGGTAAAAGGACAGGGC |
